# Unraveling structural and conformational dynamics of *DGAT1* missense nsSNPs in dairy cattle

**DOI:** 10.1038/s41598-022-08833-6

**Published:** 2022-03-22

**Authors:** Rajesh Kumar Pathak, Byeonghwi Lim, Yejee Park, Jun-Mo Kim

**Affiliations:** Department of Animal Science and Technology, Chung-Ang University, Anseong-si, Gyeonggi-do 17546 Republic of Korea

**Keywords:** Computational biology and bioinformatics, Systems biology

## Abstract

Cattle are domestic animals that have been nourishing humans for thousands of years. Milk from cattle represents a key source of high-quality protein, fat, and other nutrients. The nutritional value of milk and dairy products is closely associated with the fat content, providing up to 30% of the total fat consumed in the human diet. The fat content in cattle milk represents a major concern for the scientific community due to its association with human health. The relationship between milk fat content and diacylglycerol o-acyltransferase 1 gene (*DGAT1*) is well described in literature. Several studies demonstrated the difference in fat contents and other milk production traits in a wide range of cattle breeds, to be associated with missense non-synonymous single nucleotide polymorphisms (nsSNPs) of the *DGAT1* gene. As a result, an nsSNPs analysis is crucial for unraveling the DGAT1 structural and conformational dynamics linked to milk fat content. DGAT1-nsSNPs are yet to be studied in terms of their structural and functional impact. Therefore, state-of-the-art computational and structural genomic methods were used to analyze five selected variants (W128R, W214R, C215G, P245R, and W459G), along with the wild type DGAT1. Significant structural and conformational changes in the variants were observed. We illustrate how single amino acid substitutions affect DGAT1 function, how this contributes to our understanding of the molecular basis of variations in DGAT1, and ultimately its impact in improving fat quality in milk.

## Introduction

Cattle milk is the most-consumed milk worldwide and is recognized as a nutritionally superior food due to its capacity to satisfy nutritional and physiological requirements^1^. It has a high calcium, magnesium, and potassium content, minerals thought to be under-consumed in adult diets^2^. Additionally, it contains several water-soluble vitamins and is a good source of thiamine (B1), riboflavin (B2), and cobalamin (B12). Vitamins A and D can be found in high concentrations in milk, depending on the fat level^2^. Milk fats, which are primarily made up of triglycerides (one molecule of glycerin and three fatty acids), are one of the most important indicators of milk quality. It is vital in conferring microbial resistance and hence preserving animal homeostasis^3^. The nutritional value of milk and dairy products is closely associated with the fat content of milk, making up approximately 30% of the total fat consumed in the human diet^4^. Several studies have shown the potential impact of dietary fatty acids on human nutrition and recommended limits for dietary fat consumption^5–7^. Milk fat is made up of a complex mixture of lipids, which is composed of approximately 70% saturated fatty acids and 30% unsaturated fatty acids. While some fatty acids such as phospholipids are crucial for human health and nutrition, they are also essential constituents of cell membranes^8^. However, the high content of saturated fatty acids in milk is generally undesirable because it is associated with a variety of illnesses; including obesity, elevated blood cholesterol, and cardiovascular-related problems^7,9^.

**Figure 1:**
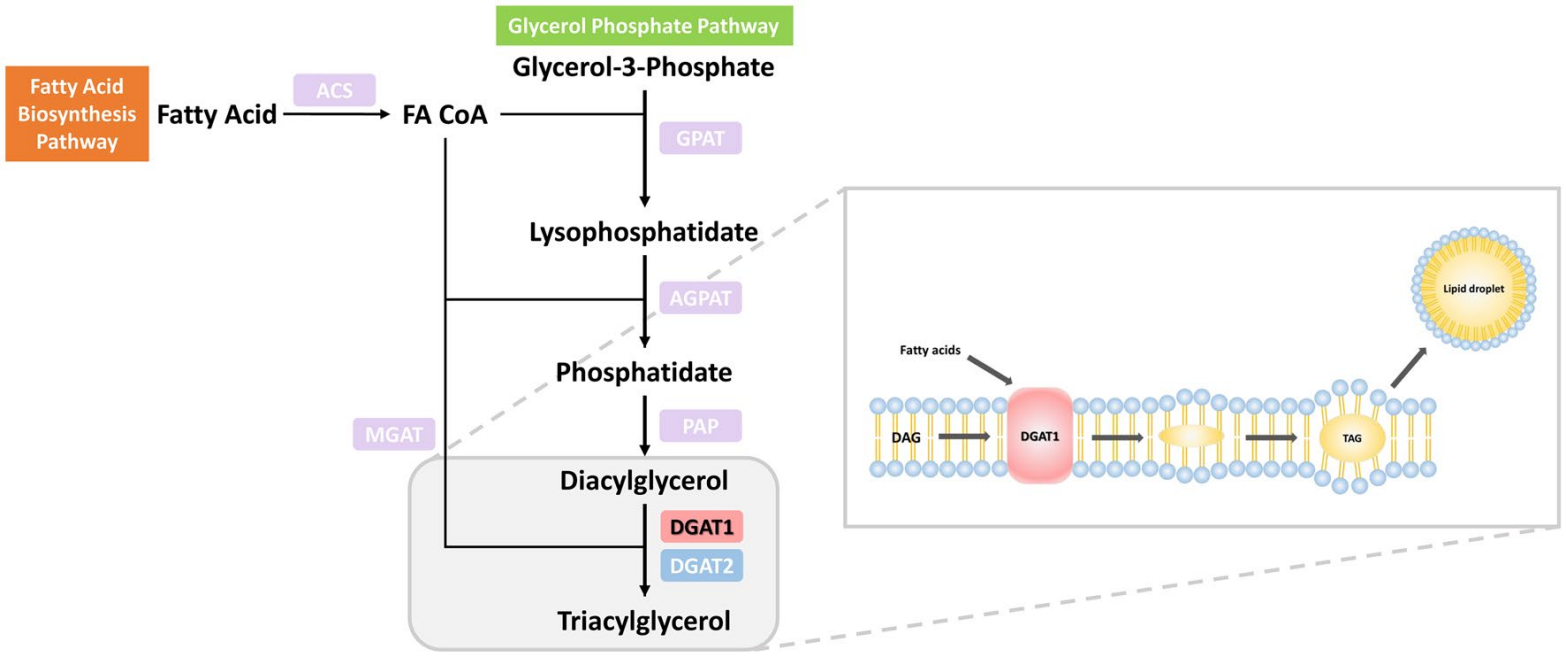
Schematic pathway of triacylglycerol biosynthesis. It illustrates the role of DGAT1 in triacylglycerol synthesis that is responsible for fat content in the milk.

The search for candidate genes that improve milk quality, particularly its fat content, has become increasingly important in recent years (Fig. 1). Most key traits of farm animals have economic importance and are associated with polygenes positioned at quantitative trait loci (QTLs), dispersed throughout the genome. Initial research identified the approximate positions of milk-related QTLs, including one controlling milk fat content at BTA14's proximal 3-cm marker interval^3,10–12^. The diacylglycerol o-acyltransferase 1 gene (DGAT1) has been investigated as a key candidate and functional gene for fat trait and milk production^11,13^. It is found in the centromeric region of *Bos taurus* autosome 14 and encodes acyl-coenzyme A:diacylglycerol acyltransferase, a protein implicated in fat metabolism in cattle breeds^11,13,14^. Genome-wide association studies (GWAS) identified several single nucleotide polymorphisms (SNPs) and their association with milk fat trait(s) of interest by using linkage disequilibrium that exists among causative variants, and one or more genetic marker^15^. All the missense variants or non-synonymous SNPs (nsSNPs) are not structurally or functionally affecting; however, numerous deleterious variants can affect the physiological system of an organism. Furthermore, experimental studies have suggested that one-third of non-synonymous variants are deleterious^16^. Several reports spanning a wide range of cattle breeds investigated the impact of the K232A variant on milk fat content and composition, as well as other milk production traits^17^. Nevertheless, a computational investigation of missense variants of the DGAT1 and their crucial role in milk fat is currently lacking from the literature. As a result, we set out to undertake a complete in silico examination of the impact of DGAT1 variants linked to milk production in dairy cattle. We started with a list of 73 missense variants collected from the Ensembl using the BioMart data mining tool^18,19^. Finally, five variants of the DGAT1 protein, W128R, W214R, C215G, P245R, and W459G, were chosen and analyzed in detail utilizing all-atom molecular dynamics (MD) simulations for 100 ns to examine their temporal evolution impact. Our study provides novel insights into our understanding of the structural and conformational dynamics of wild type as well as DGAT1 variants and their role in improving milk quality in the future.

## Results

### Screening of significant nsSNPs

A total of 73 nsSNPs predicted by SIFT were first analyzed to identify the ones that might have a significant influence (Supplementary Table S1). Out of the 73 nsSNPs, 5 were considered deleterious based on PROVEAN prediction. An nsSNP with a PROVEAN score of < − 2.5 was regarded as deleterious, while nsSNPs with a score > − 2.5 were considered neutral. PROVEAN predicted that 64 of the 73 nsSNPs would be deleterious while the remaining 9 would be neutral. Of the 64 PROVEAN predicted nsSNPs, 5 nsSNPs (W128R, W214R, C215G, P245R, and W459G) were found significant based on their lowest score (Table 1). The PROVEAN score for 5 shorted nsSNPs ranged between − 8.543 and − 11.385.The high deleterious nature of these selected nsSNPs was also confirmed by different other sequence-based tools including PredictSNP, MAPP, PhD-SNP, PolyPhen-1, PolyPhen-2, and SNAP (Supplementary Table S2).

**Table 1:** A list of five selected missense nsSNPs predicted as deleterious by different sequence based tools. SIFT, PROVEAN, PredictSNP, MAPP, PhD-SNP, PolyPhen-1, PolyPhen-2, and SNAP were employed.

S.N.	SNP ID	Location	Variant alleles	Substitution	Overall prediction
1	rs466331549	14:610211	T/C	W128R	Deleterious
2	rs451872523	14:610865	T/C	W214R	Deleterious
3	rs440590330	14:610868	T/A/G	C215G	Deleterious
4	rs456383648	14:611059	C/G	P245R	Deleterious
5	rs474703848	14:612411	T/G	W459G	Deleterious

### Structural modeling, analysis and visualization of DGAT1 and its screened nsSNPs

**Figure 2:**
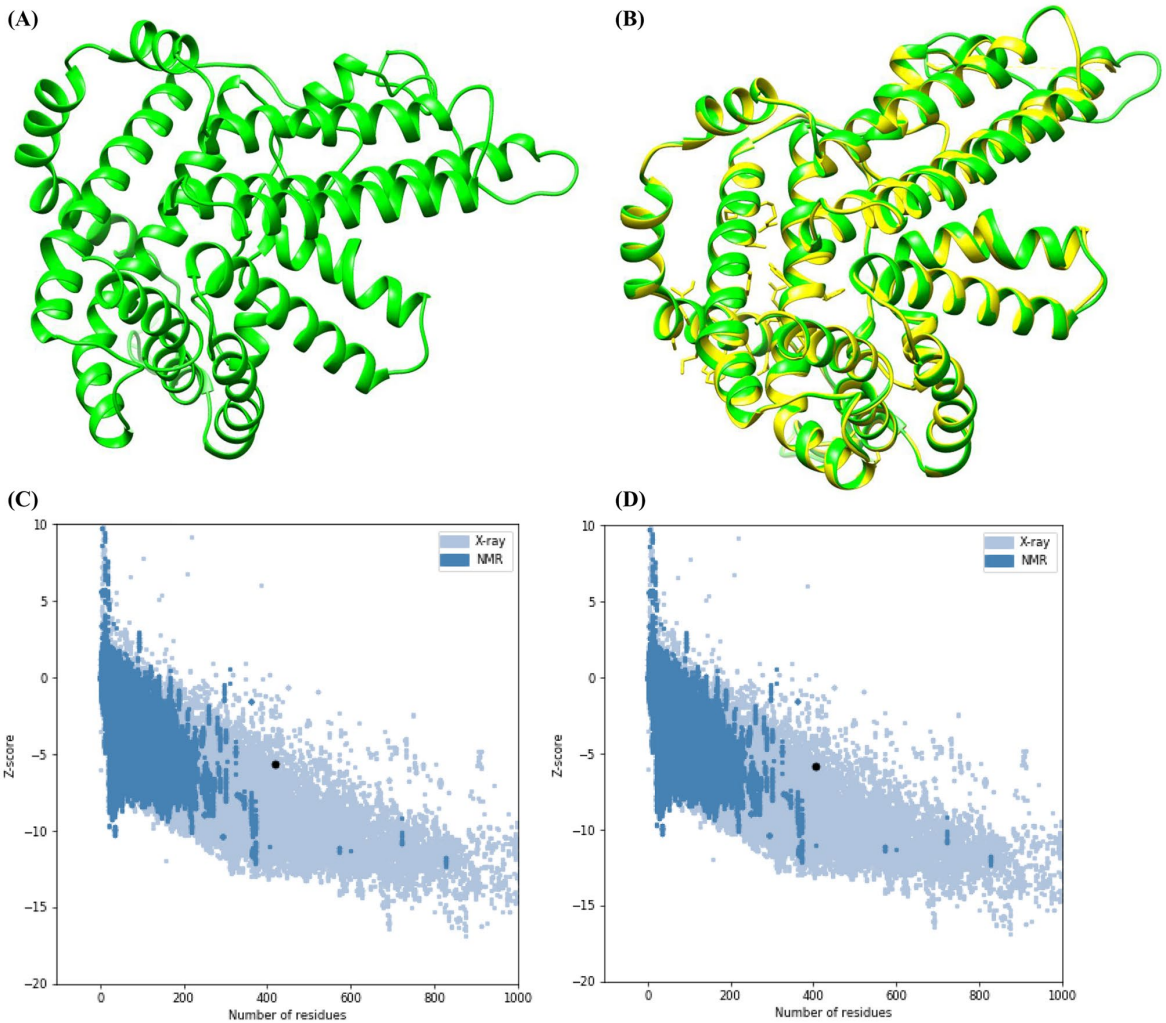
(**A**) 3D model of DGAT1. (**B**) Superimposed structure of DGAT1 with its template structure 6VYI_A. (**C**) Overall quality of DGAT1 model depicting a z-score of –5.63 (Native conformation to its template). (**D**) Overall quality of template (6VYI_A) depicting a z-score of -5.83.

SwissModel was used to generate the 3D structure of DGAT1 protein. UCSC Chimera was used to visualize the predicted model (Fig. 2A). The SPDB Viewer and the steepest descent approach were used to remove the bad contract between protein atoms and stabilize the stereochemical properties of the DGAT1 model through energy minimization. Furthermore, the DGAT1 model stability was verified using the SAVES server, while the model's reliability of the backbone of torsion angles was assessed using PROCHECK. The latter computes the amino acid residues that fall within the Ramachandran plot's existing zones. For the DGAT1 model, the Ramachandran plot analysis revealed that 86.9% of residues fell in the most favored regions, 12.3% in additional allowed regions, 0.8% in generously allowed regions, and none fell in disallowed regions. ProSA also confirmed the DGAT1 model's reliability. The model's quality was further assessed by superimposing the predicted structure over the experimentally obtained structure and calculating the atoms RMSD using UCSF Chimera. This revealed that the predicted model is good and quite similar to the template (Fig. 2B). The model's energy profile and Z-score value were calculated using a distance-based pair potential, which determines the interaction energy per residue. The modeled DGAT1 had a Z-score of − 5.63, and the template had a Z-score of − 5.83; thus, indicating the good quality of the model (where the energy of ProSA in negative reflects the model's reliability) (Fig. 2C,D). Further, mutagenesis wizard from PyMOL was used for generating the 3D model of the W128R, W214R, C215G, P245R, and W459G variants of DGAT1 protein structure. The mutagenesis wizard can be used to make amino acid residues mutation/changes in the 3D structure of a protein at any position.

### Evaluating the structural effects of deleterious nsSNPs of DGAT1

Structure-based tools were used to screen modeled structure of DGAT1 variants W128R, W214R, C215G, P245R, and W459G for their structural impact. DynaMut, CUPSAT, and I-mutant were used for this purpose. DynaMut uses a consensus-based technique centered on protein motion and graph-based signatures to estimate the effects of missense variations on protein stability. CUPSAT assesses the amino acid environment of the mutation site using amino acid atom potentials and torsion angles to predict protein stability changes due to point mutations. Lastly, I-mutant uses SVM-based predictors to estimate how a protein's stability changes as a result of variations. Based on the change in Gibbs free energy value, it can be determined whether a mutation would mostly destabilize, largely stabilize, or have a weak influence on the protein. Based on the comparative analysis of these tools, it was predicted that the selected nsSNPs are destabilizing the structure of DGAT1 protein, which might ultimately affect milk production and fat content. The results are shown in Table 2.

**Table 2:** List of selected significant deleterious nsSNPs and their effects on DGAT1 structure predicted by structure-based tools.

SNP ID	Variant	DynaMut	CUPSAT	I-mutant
rs466331549	W128R	Destabilizing	Stabilizing	Decrease
rs451872523	W214R	Destabilizing	Destabilizing	Decrease
rs440590330	C215G	Destabilizing	Destabilizing	Decrease
rs456383648	P245R	Stabilizing	Destabilizing	Decrease
rs474703848	W459G	Destabilizing	Destabilizing	Decrease

**Figure 3:**
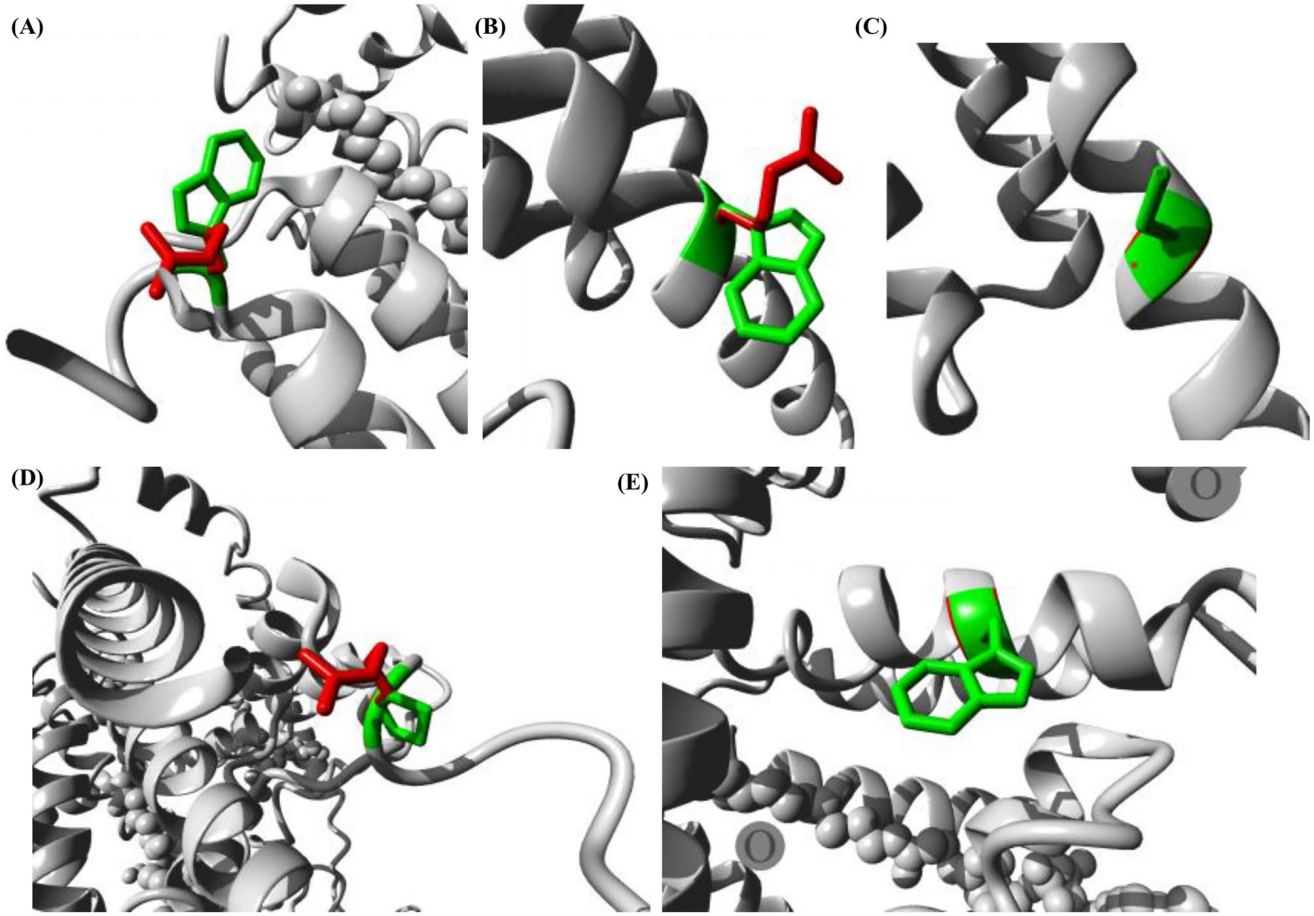
Close-up view of wild type (green) and variant (red) amino acid residues of selected deleterious nsSNPs visualized using the HOPE server. (**A**) W128R, (**B**) W214R, (**C**) C215G, (**D**) P245R, and (**E**) W459G.

Further, these DGAT1 nsSNPs were visualized structurally using the HOPE server (Fig. 3). Table 3 shows the structural effects of these five deleterious variants. All variant residues, except P245R, were smaller than the wild type residues. As the mutant residue arginine is larger than the wild type residue proline, for the P245R nsSNP residue it was predicted that it would not fit in the core of the protein. Furthermore, arginine is positively charged while the wild type residue at 245 proline is neutral, which may cause problems during protein folding.

**Table 3:** Structural impacts of DGAT1 variants due to amino acid changes.

Variant	Structural change	Properties of amino acid	Domain
W128R W214R		The mutant residue is smaller than the wild type residue. The wild type residue charge is neutral while the mutant residue charge is positive. The wild type residue is more hydrophobic than the mutant residue	The mutated residue is located in a domain that is important for the main activity of the protein. Mutation of the residue might disturb protein function
C215G		The mutant residue is smaller than the wild type residue. The wild type residue is more hydrophobic than the mutant residue	The mutated residue is located in a domain that is important for the main activity of the protein. Mutation of the residue might disturb protein function
P245R		The mutant residue is bigger than the wild type residue. The wild type residue charge is neutral, while the mutant residue charge is positive. The wild type residue is more hydrophobic than the mutant residue	The mutated residue is located in a domain that is important for the main activity of the protein. Mutation of the residue might disturb protein function
W459G		The mutant residue is smaller than the wild type residue. The wild type residue is more hydrophobic than the mutant residue	The mutated residue is located in a domain that is important for the main activity of the protein. Mutation of the residue might disturb protein function

### Conservation analysis and identification of functional regions in DGAT1

**Figure 4:**
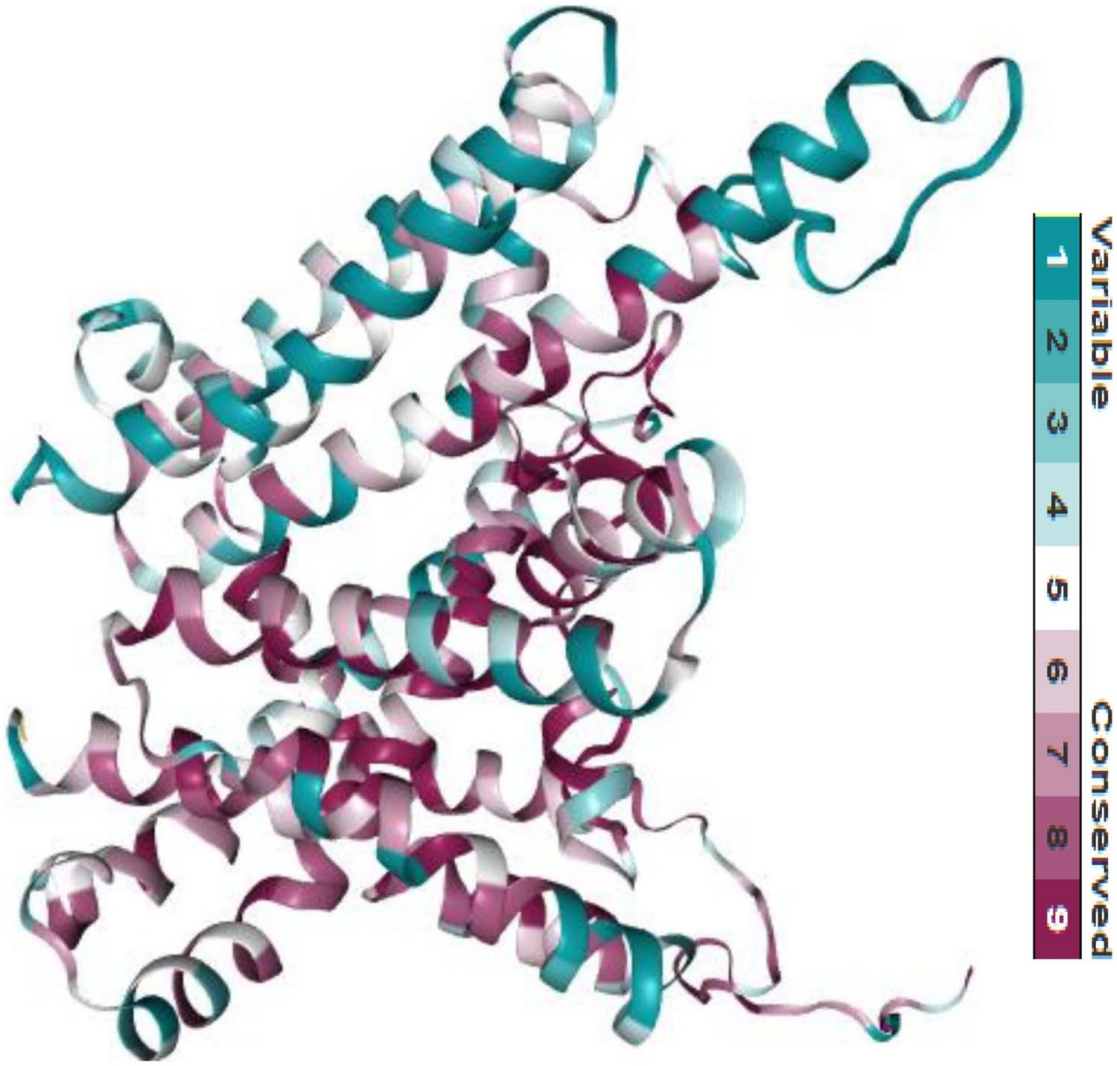
DGAT1 conservation analysis performed using ConSurf and depicting variable as well as conserved residues (score from 1 to 9 based on color).

The level of residue conservation offers an approximate of the structural and functional impact of any protein due to variations. In nature, a deleterious variant of a highly conserved residue will almost certainly be damaging. The 3D structure of DGAT1 was used as an input query to determine the conservation level based on the score predicted by the ConSurf server (Fig. 4). The ConSurf results demonstrated that two of the selected variants, W214R and W459G, were highly conserved obtaining a conservation score of 9. The conservation scores of other selected variants, W128R, C215G, and P245R were 7, 6, and 6, respectively. However, the distribution of conserved residues was random. The position from 252 to 479 was predicted to be an area that contain numerous conserved residues. Furthermore, average variability and conservation residues (score of 5) were also found among this region. This analysis provides a better understanding of the functional regions in DGAT1 and their impact on milk production. Since the selected variants are found in conserved regions they might influence the fat content of milk.

### MD simulation analysis for decoding the complex nature of DGAT1 wild type and its variants

**Figure 5:**
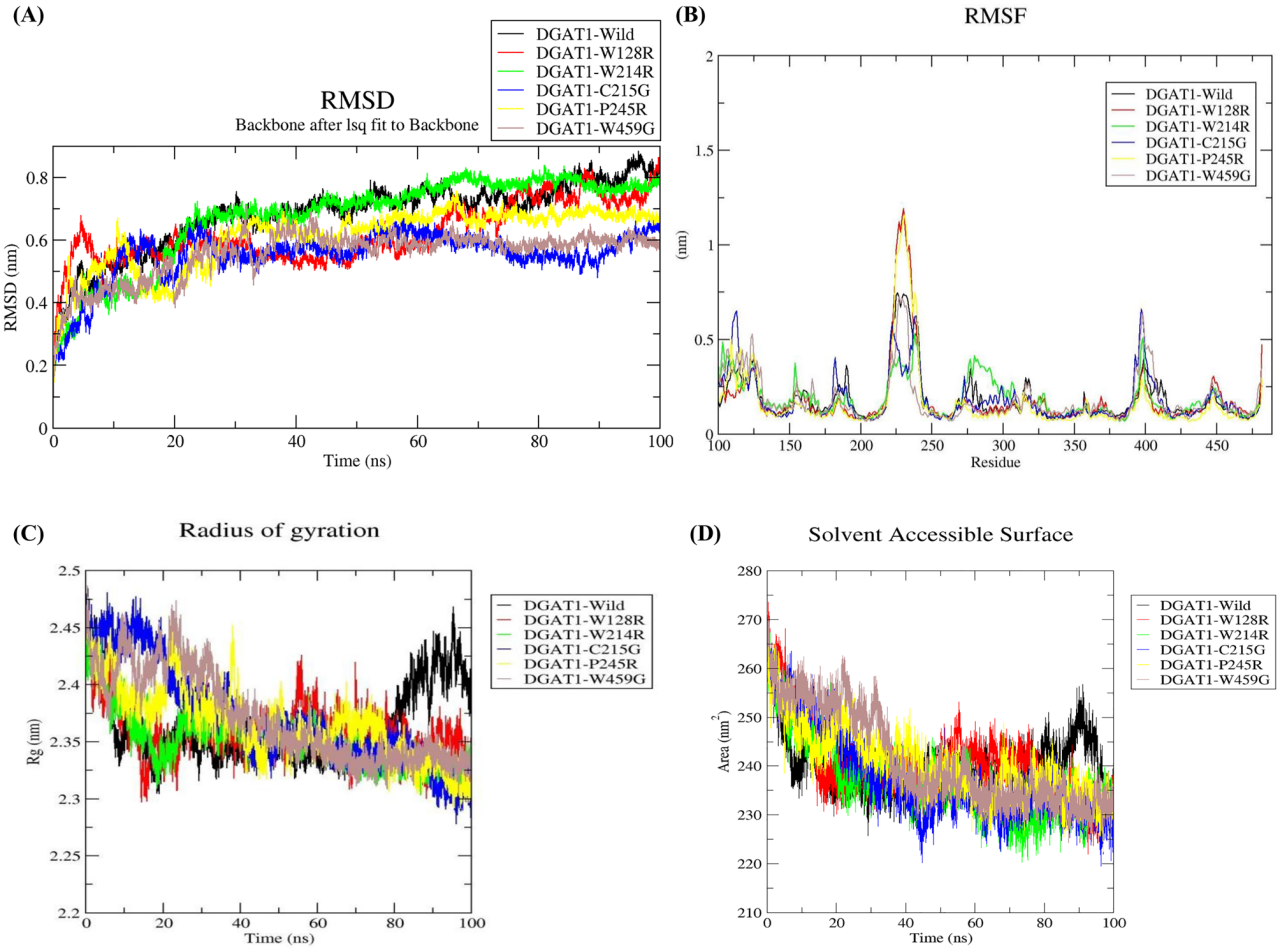
Conformational dynamic analysis of DGAT1-wild type and its variants using MD simulation. (**A**) RMSD, (**B**) RMSF, (**C**) Rg, and (**D**) SASA.

The conformational differences between the wild type and variant proteins were decoded using MD simulations. An MD simulation of 100 ns was performed using Gromacs after generating six systems (DGAT1-wild-type, DGAT1-W128R, DGAT1-W214R, DGAT1-C215G, DGAT1-P245R, and DGAT1-W459G). For trajectory analysis, Gromacs utilities were employed (Fig. 5).

#### Analysis of conformational stability

The wild type and variant DGAT1 proteins' RMSDs were calculated to predict their respective stabilities. The RMSD discrepancies created during MD simulation can be used to measure the protein's stability in relation to its structure^20,21^. The RMSD deviations observed during the MD simulation can be used to measure the protein's stability in relation to its structure. Smaller RMSD deviations indicate a more stable protein structure. The backbone RMSD was plotted against time to measure structural conformational variations (Fig. 5A).The RMSD of the DGAT1-wild type averaged 0.68 nm. Whereas the RMSD values of the variants DGAT1-W128R, DGAT1-W214R, DGAT1-C215G, DGAT1-P245R, and DGAT1-W459G were 0.62, 0.67, 0.55, 0.61, and 0.55 nm, respectively.

#### Analysis of structural flexibility

We computed structural flexibility for all variants, including the wild type structure, after assessing the RMSD. In nature, protein structures are dynamic, requiring structural flexibility to maintain their properties^22^. Figure 5B shows a plot of RMSF values against all residues. The average predicted RMSF value of DGAT1-wild type was 0.20 nm and for variants DGAT1-W128R, DGAT1-W214R, DGAT1-C215G, DGAT1-P245R, and DGAT1-W459G were 0.21, 0.22, 0.20, 0.18, and 0.20 nm respectively.

#### Analysis of structural compactness

The Rg values of all six systems were used to examine the structural compactness of the DGAT1 structure before and after generating variations. The mechanism of structural compactness, stability, and folding of a protein structure can be derived using the time evolution of Rg values^23^. We calculated and plotted the Rg values of DGAT1-wild type, DGAT1-W128R, DGAT1-W214R, DGAT1-C215G, DGAT1-P245R, and DGAT1-W459G systems from the MD trajectories. The average Rg were 2.36, 2.36, 2.35, 2.37, 2.36, and 2.37 nm, respectively (Fig. 5C).

#### Analysis of solvent accessible surface area

The average SASA values for DGAT1-wild type, DGAT1-W128R, DGAT1-W214R, DGAT1-C215G, DGAT1-P245R, and DGAT1-W459G were 240, 240, 236, 237, 240, and 241 nm^2^, respectively and were plotted over 100 ns of MD simulation (Fig. 5D). The variation in the SASA values of W214R and C215G signified the folding nature of DGAT1 structure upon change in amino acid residue. The other variant showed similar conformational stability to the wild type.

#### Analysis of intramolecular hydrogen bond

**Figure 6:**
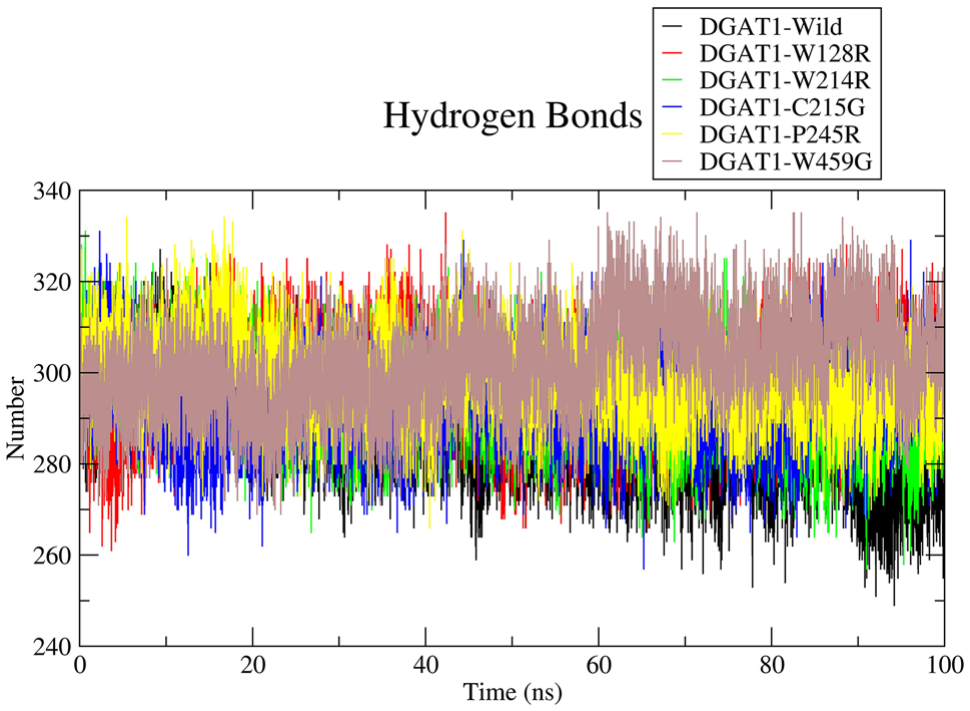
Intramolecular hydrogen bonds calculation illustrated as a time-dependent change. DGAT-Wild (black), DGAT1-W128R (red), DGAT1-W214R (green), DGAT1-C215G (blue), DGAT1-P245R (yellow), and DGAT1-W459G (brown).

In this study, the number of intramolecular hydrogen bonds was used to predict the effect of variants on the DGAT1 protein structure. Figure 6 shows the number of hydrogen bonds formed by the wild type and variant proteins as a function of time. The average number of intramolecular hydrogen bonds for DGAT1-wild type, DGAT1-W128R, DGAT1-W214R, DGAT1-C215G, DGAT1-P245R, and DGAT1-W459G were 287, 297, 294, 294, 299, and 301, respectively.

#### Principal component analyses

**Figure 7:**
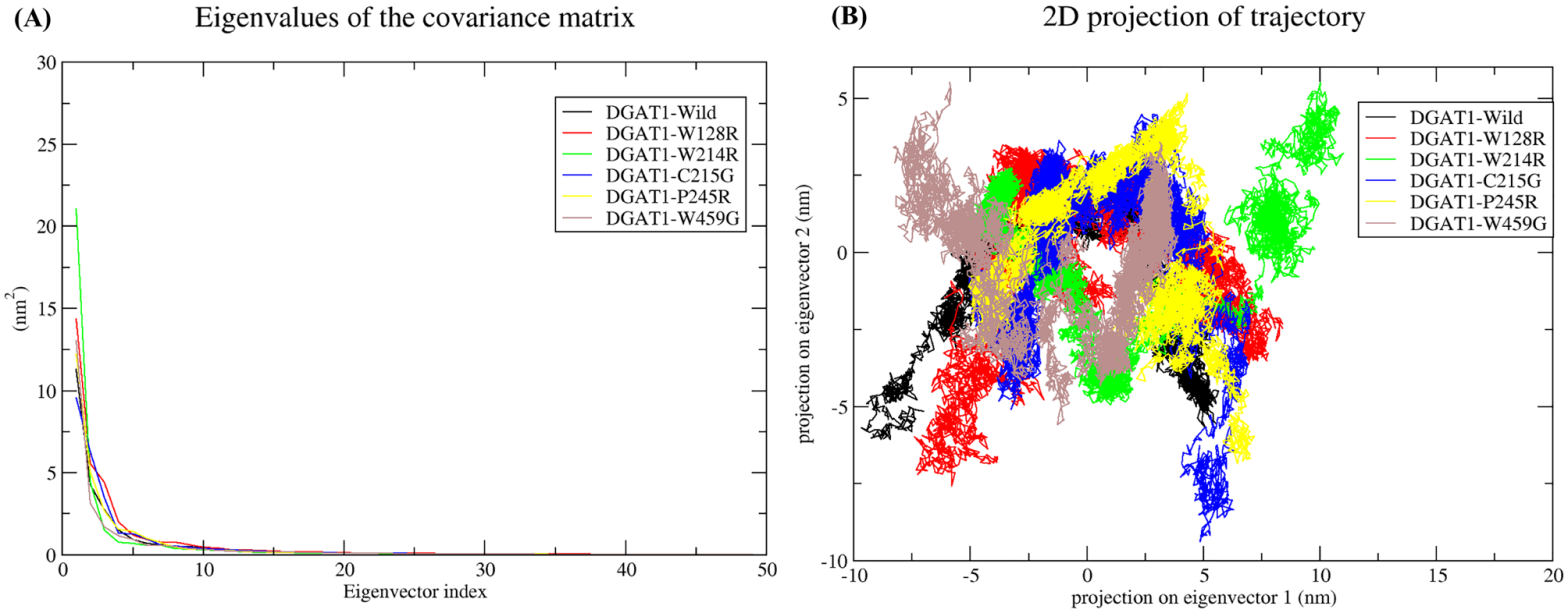
Principal component analysis using molecular dynamics simulation data. (**A**) First 50 eigenvectors against eigenvalue of the DGAT1-wild type and its variants. (**B**) First two eigenvectors depicting the projection of protein motion in phase space for DGAT1-wild type and its variants.

Principal component analysis (PCA) was employed to visualize the protein global motions by using a small number of principal motions, which are defined by eigenvectors and eigenvalues. We examined whether the first few principal motions or eigenvectors playing a key role in the protein motions. Therefore, first 50 eigenvectors against eigenvalue of the DGAT1-wild type and its variant proteins were plotted in Fig. 7A. The percentage of motion for wild type and variants was computed. The first 5 principal components accounted for 75.84% of the DGAT1-wild type protein motion, and 78.34%, 84.93%, 77.31%, 79.39%, and 78.26% of DGAT1-W128R, DGAT1-W214R, DGAT1-C215G, DGAT1-P245R, and DGAT1-W459G protein motion, respectively. Additionally, a 2D plot for assessing the wild type and variant proteins’ dynamics was produced. We imagine a cluster of stable states in wild type and variants based on PC1 and PC2 projection (Fig. 7B).

### Analysis of PPIs network for visualizing interacting partners of DGAT1

**Figure 8:**
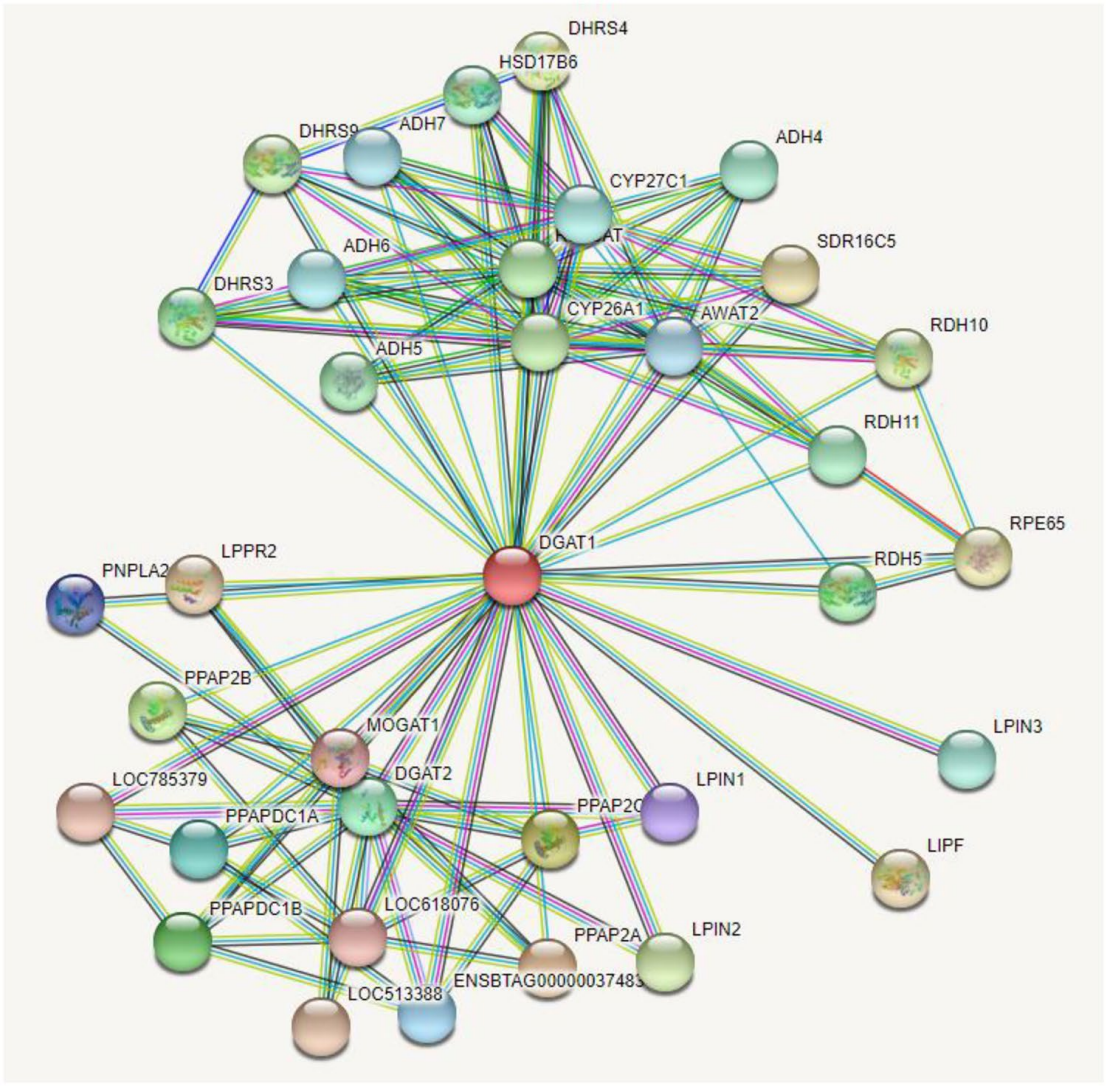
Protein–protein interactions (PPIs) network generated by STRING depicting the interacting partners of DGAT1; Red color node represent query protein, filled nodes have known 3D structure and empty nodes represent proteins of unknown 3D structure. Besides, pink color edges represent experimentally determined interaction, green-neighborhood, red-gene fusion, blue-gene co-occurrence, sky blue-database curated, dark-coexpression, kelly-text mining and purple colour edges represent protein homology based interactions.

The PPIs network construction and analysis based on highest confidence score using the STRING database revealed that the DGAT1 network has 35 nodes, 122 edges, and an average predicted node degree of 6.97 (Fig. 8). The predicted interacting partners of DGAT1 with their respective scores are provided in Supplementary Table S3. These proteins work along with DGAT1 to perform common tasks. The analysis predicted that the network is mostly involved in different biological processes, which includes retinal metabolic process (GO:0042574), Monoacylglycerol biosynthetic process (GO:0006640), Retinoic acid biosynthetic process (GO:0002138),Triglyceride biosynthetic process (GO:0019432), Diacylglycerol biosynthetic process (GO:0006651), Sphingosine metabolic process (GO:0006670), Retinol metabolic process (GO:0042572), Acylglycerol biosynthetic process (GO:0046463), Retinoid metabolic process (GO:0001523), Terpenoid biosynthetic process (GO:0016114), Phospholipid dephosphorylation (GO:0046839), Primary alcohol metabolic process (GO:0034308), Triglyceride metabolic process (GO:0006641), Cellular hormone metabolic process (GO:0034754), Olefinic compound metabolic process (GO:0120254), Cellular aldehyde metabolic process (GO:0006081), Polyol metabolic process (GO:0019751), Alcohol metabolic process (GO:0006066), Lipid modification (GO:0030258), Cellular lipid metabolic process (GO:0044255), Lipid biosynthetic process (GO:0008610), Lipid metabolic process (GO:0006629), Small molecule metabolic process (GO:0044281), Regulation of biological quality (GO:0065008), and Metabolic process (GO:0008152).

## Discussion

Milk from cattle is an essential part of the human diet. Its impact on health is influenced by a variety of elements, including its fat composition. Fatty acids in milk come from two sources; they transfer from the blood via milk-producing cells and synthesize de novo in milk-producing cells^24,25^. Successful lactation is controlled and regulated by the metabolism of adipose tissues. The metabolism in adipose tissues of dairy cattle has been well-documented in the scientific literature^26,27^. Previous studies revealed alterations in enzymes that controlled the metabolism of lipid in adipose tissues during lactation^28,29^. These investigations were followed up by other researchers and found that the adaptation of metabolic activities is managed by many endocrine and neurocrine systems, which include growth hormone, insulin, and the sympathetic nervous system^27,30^. When compared to ordinary or lower genetic merit cattle, these studies found and consistently demonstrated that dairy cattle’s with high genetic merit for milk production had higher rates of subcutaneous adipose tissue lipolysis and lower rates of lipogenesis^27,31,32^. Therefore, adipose tissue is important for the success and efficiency of lactation, and recent research has produced various data sets on genetic differences and changes in adipose tissue gene transcription in dairy cattle^27^.

*DGAT1* is regarded as one of the essential components in the metabolism of glycerol lipids and is involved in the final phase of triglyceride synthesis in adipocytes. Previous studies confirmed its role in maintaining the fat content and composition of fatty acids in milk^3^. Fatty acid concentration is known to be regulated by a variety of physiological and environmental variables. Many studies have shown that concentrations change during lactation^25^. A previous study demonstrated that the fatty acid profile is significantly affected during the lactation stage, it was found that only two were unaffected out of 40 analyzed^33^. Besides, another research investigated that lactation phase affected all fatty acid groups, but in different ways due to different SNPs^25^. The K231A, *DGAT1* variant is responsible for a change in fat and protein percentage during lactation and is associated with the highest reduction of fat percentage^34^. Therefore, *DGAT1* is documented as one of the key candidate genes for maintaining fat quality in milk. In recent years, this gene attracted researchers due to its importance in producing healthy milk, and its nsSNPs are liable for milk quality issues. In the present study, we tried to predict the conformational and structural dynamics of *DGAT1* and its missense nsSNPs through structural genomics approaches for its possible utilization in research associated with milk production.

The current work was primarily interested in nsSNPs and their regulatory role in milk production. Therefore, only nsSNPs were investigated, and their structural as well as functional effects on the DGAT1 protein were predicted. The deleterious nature of selected nsSNPs was analysed by various sequence and structure-based tools. Based on the obtained score, 5 selected significant nsSNPs i.e. W128R, W214R, C215G, P245R, and W459G along with wild-type structure were further evaluated through structural modeling and molecular dynamics simulation, followed by network analysis. Based on RMSD results, the variants seemed to be more stable than the DGAT1-wild type conformation. Therefore, it was concluded that the increased stability could have a deleterious impact on overall function of DGAT1. More research is needed to clarify why enhanced stability can be associated with a deleterious effect.

During structural flexibility analysis through RMSF, minimal fluctuations were observed in P245R, whereas other systems showed similar type of behaviour, besides high pick was observed for the W128R and P245R between residues 225–250 due to variations in amino acid residue compared to the wild type structure. During Rg analysis, the DGAT1-wild type showed an Rg peak of 2.47 after 90 ns, which was achieved 40 ns after the peaks obtained for the other variants. Furthermore, the DGAT1-C215G and DGAT1-W459G variants average Rg values were predicted to be higher than the others, indicating less compactness and more flexibility.

To define the area accessible for solvent, SASA analysis was conducted. Higher SASA values represent a unstable conformation of the protein, while lower SASA values represent conformationally stable protein^35^. During analysis, it was observed that the SASA values of W214R and C215G varied, indicating the folding nature of the DGAT1 structure as amino acid residues were changed. In addition, when compared to wild type, other variants demonstrated similar conformational stability. The protein stability is sustained by hydrogen bonding. A more stable structure has more hydrogen bonds, while the least stable structure has fewer hydrogen bonds^36^. The loss or gain of hydrogen bonds caused by deleterious nsSNPs can influence the structure and function of the protein^36^. Due to the gain of hydrogen bonds, the variants' stability was enhanced. This was in line with the results obtained following the RMSD, RMSF, and Rg analyses.

In general, proteins carry out their unique tasks through a series of atomic motions. As a result, the collective atomic motion of a target protein is utilized as a parameter to determine its stability^23,37^. Therefore, PCA analysis was conducted; the results showed that the variants cover a wide range of phase spaces when compared to the wild type. The significant increase in total motion might transform the protein function. Overall, compared to the wild type protein, high correlated motions were observed in variants. Therefore, the conformational behavior of variants was altered due to amino acid change.

Network analysis has been promoted as one of the most effective methods for decoding the key regulatory elements in biological systems and their intricate molecular machinery^38,39^. Here, the purpose of network analysis was to identify an association of DGAT1 with other proteins in a holistic view based on the information available in the STRING database. The results of network analysis clearly demonstrated the interacting partners of DGAT1 and their involvement in different biological processes. Therefore, it was concluded that DGAT1 variants might affect several biological processes, since it might be working as a master regulator.

## Materials and methods

### Dataset collection

The missense variants of the DGAT1 gene were collected from Ensembl (https://asia.ensembl.org/index.html) using the BioMart^18^ data mining tool^19^. Based on Sorting Intolerant from Tolerant (SIFT) predictions, a total of 73 nsSNPs were analyzed initially^40^. The amino acid sequence of DGAT1 was retrieved from UniProt (https://www.uniprot.org/) database (UniProt ID: Q8MK44) in fasta format^41^.

### Prediction of deleterious nsSNPs through sequence-based tools

Several standard tools were used to predict the functional effect of nsSNPs on the protein. Based on reliable prediction using all employed tools, nsSNPs were classified as deleterious. SIFT (https://sift.bii.a-star.edu.sg/) and Protein Variation Effect Analyzer (PROVEAN: http://provean.jcvi.org/index.php) was used to estimate the effect of amino acid substitution on protein structure and function^40,42^. Furthermore, PredictSNP^43^ (https://loschmidt.chemi.muni.cz/predictsnp/) that access different other tools related to SNP prediction such as MAPP, PhD-SNP, PolyPhen-1, PolyPhen-2, and SNAP was used to evaluate the nature of variants^44–48^.

### Molecular modeling of DGAT1 and its variants

The DGAT1 target protein sequence was compared to the PDB database (https://www.rcsb.org/) using NCBI BLASTp^49^ to find a suitable template and decide the 3D modelling strategy. Further, SwissModel (https://swissmodel.expasy.org/interactive) was used to model the wild type DGAT1 3D structure, 6VYI chain A was used as a template^50^. SPDB viewer was used to refine the model through energy minimization^51^. The Ramachandran plot of the predicted model was examined using the PROCHECK^52^ from the Structural analysis and verification (SAVES) server (https://saves.mbi.ucla.edu/). Protein structure analysis (ProSA) was used to determine the overall quality of the model (https://prosa.services.came.sbg.ac.at/prosa.php)^53^. UCSF Chimera was used to visualize and superimpose the DGAT1 model with its structural homolog 6VYI_A^54^. The amino acid variations W128R, W214R, C215G, P245R, and W459G were structurally incorporated into the DGAT1 model using the PyMOL software's mutagenesis wizard (https://pymol.org/2/).

### Prediction of effects of missense variant on protein through structure-based tools

The three structure-based methods were used to investigate the changes in protein stability induced by deleterious predicted nsSNPs. DynaMut (http://biosig.unimelb.edu.au/dynamut/), CUPSAT (http://cupsat.tu-bs.de/), and I-Mutant (http://gpcr2.biocomp.unibo.it/cgi/predictors/I-Mutant3.0/I-Mutant3.0.cgi) predicted the influence of nsSNPs on protein stability and dynamics^55–57^. Further, The Have Your Protein Explained (HOPE) server (https://www3.cmbi.umcn.nl/hope/) was used to visualize and analyze the variants^58^.

### Prediction of functional and conserved regions

The evolutionary conservation of a protein or a nucleic acid demonstrates a balance between the macromolecule's natural tendency to mutate and the overall need to maintain structural integrity and function. Here, the ConSurf (https://consurf.tau.ac.il/) server was used to predict the evolutionarily conserved amino acid residues sites of DGAT1. A 3D structure of DGAT1 was used as an input query to determine the conservation score. The conservation score ranges from 1 to 9, with 1 indicating that the residue is highly changeable and 9 indicating that it is well conserved^59^.

### Molecular dynamics (MD) simulation

To explore the structural impact of DGAT1 protein due to the variation, all-atom MD simulations were performed using GROMACS 2018.1^60^. All simulations were run within the High-Performance computing (HPC) facility using a GPU node. The topology of the wild type and DGAT1 variants was generated using the Amber99sb-ildn force field^61^. The systems were neutralised and subjected to the steepest energy minimization to achieve a maximal force below 1000 kJ/mol/nm; thus, reducing steric hindrance. The particle mesh Ewald (PME) approach was used to determine long-range electrostatic interactions^62^. We employed a radius cut-off of 1.0 nm for Lennard–Jones and Coulomb interactions, and the LINCS algorithm for H-bond length constrains^63^. Long-range electrostatics was predicted using the PME approach with 1.6-Å Fourier grid spacing, while short-range non-bonded interactions were predicted using a 10-Å cut-off distance. All bonds, including H-bonds, were fixed using shake algorithms. Systems were equilibrated following energy minimization, and then position-restraint simulations were run under NVT and NPT conditions to maintain the volume, temperature, and pressure^64^. The final MD simulation was carried for 100 ns. Root–mean–square deviation (RMSD), root–mean–square fluctuation (RMSF), radius of gyration (Rg), solvent-accessible surface area (SASA), hydrogen bonds, and principal component analysis (PCA) were calculated to predict the conformational and structural behaviour of DGAT1 and its variants; Gromacs gmx ‘rms’, ‘rmsf’, ‘gyrate’, ‘sasa’, ‘hbond’, and ‘covar’ were used to analyze and visualize the generated files (https://plasma-gate.weizmann.ac.il/Grace/).

### Prediction of protein–protein interactions (PPIs) network

STRING (https://string-db.org/) database version: 11.5, a large collection of known and predicted protein–protein interactions, was used to analyze DGAT1 interactions. The network was generated at the highest confidence score (0.900) with no more than 50 interactors in 1st shell using *Bos taurus* database. This analysis is necessary and important in the context of this study because DGAT1 is a crucial protein linked with milk production and fat content. As, when any variations occur in this protein, its interacting partners may be affected, so that the functions that the network is involved is also affected^65^.

## Conclusion

Non-synonymous SNPs are one of the most common genetic variation linked to a variety of dysfunctions in the target gene. Analysis of nsSNPs can provide insight into their functional role and associated pathways as well as offer solution for related problems. Studying nsSNPs through experimental approaches is time consuming and costly. The computational research presented here demonstrates a quick and cost-effective method for screening deleterious nsSNPs associated with structural and conformational destabilization of the protein. The impact of amino acid substitutions of highly deleterious variants shorted amongst the 73 predicted DGAT1 variants (W128R, W214R, C215G, P245R, and W459G) were determined using sequence and structure-based tools. Finally, these variants were investigated using MD simulations of 100 ns. Furthermore, the interacting partners of DGAT1 are also predicted to investigating its role in different biological processes. MD simulation studies have revealed that the deleterious effects of these variants may be attributable to structural changes in DGAT1. Based on our RMSD, RMSF, Rg, SASA, H-bonding, and PCA results, significant conformational changes were observed due to amino acid variation. Thus, the present study provides novel insight into the impact of missense nsSNPs on the structural and conformational dynamics of DGAT1. These findings could be helpful to direct future studies in improving milk quality and fat content in dairy cattle for ensuring nutritional security of the rapidly growing world populations.

## Supplementary Information

Supplementary Tables.
