# Supplementary material for: Unraveling structural and conformational dynamics of DGAT1 missense nsSNPs in dairy cattle
Source: Sci Rep. 2022 Mar 22;12:4873. doi: 10.1038/s41598-022-08833-6 (PMC8940929; doi:10.1038/s41598-022-08833-6)
Supplement: Supplementary file 1 — Supplementary Tables. [file 41598_2022_8833_MOESM1_ESM.pdf]

Unraveling structural and conformational dynamics of *DGAT1* missense nsSNPs in dairy cattle

Rajesh Kumar Pathak, Byeonghwi Lim, Yejee Park and Jun-Mo Kim*

Department of Animal Science and Technology, Chung-Ang University, Anseong-si, Gyeonggi-do 17546, Republic of Korea

Supplementary Materials

Supplementary Table S1. List of deleterious missense variants of DGAT1 retrieved from Ensembl using BioMart.

SN	Name/SNP id	Chromosome	Start bp	End bp	Variant alleles	Variant consequence	Start aa	End aa	Protein allele	SIFT prediction	SIFT score	Biotype
1.	rs452756537	14	604225	604225	C/G	Missense variant	12	12	R/G	Deleterious	0	Protein coding
2.	rs472241168	14	604241	604241	C/G	Missense variant	17	17	S/W	Deleterious	0	Protein coding
3.	rs448355632	14	604342	604342	G/T	Missense variant	51	51	D/Y	Deleterious	0	Protein coding
4.	rs464764090	14	604379	604379	T/C	Missense variant	63	63	L/P	Deleterious	0	Protein coding
5.	rs442695761	14	608004	608004	A/G	Missense variant	66	66	H/R	Deleterious	0	Protein coding
6.	rs447545890	14	608015	608015	G/T	Missense variant	70	70	D/Y	Deleterious	0	Protein coding
7.	rs476228763	14	608074	608074	T/G	Missense variant	89	89	C/W	Deleterious	0	Protein coding
8.	rs482903905	14	610169	610169	C/A	Missense variant	114	114	P/T	Deleterious	0	Protein coding
9.	rs465361533	14	610206	610206	A/G	Missense variant	126	126	Y/C	Deleterious	0	Protein coding
10.	rs447910142	14	610210	610210	C/A	Missense variant	127	127	S/R	Deleterious	0	Protein coding

11.	rs466331549	14	610211	610211	T/C	Missense variant	128	128	W/R	Deleterious	0	Protein coding
12.	rs447276149	14	610603	610603	T/C/G	Missense variant	156	156	L/P	Deleterious	0	Protein coding
13.	rs447276149	14	610603	610603	T/C/G	Missense variant	156	156	L/R	Deleterious	0	Protein coding
14.	rs436251491	14	610618	610618	G/T	Missense variant	161	161	G/V	Deleterious	0	Protein coding
15.	rs469821622	14	610627	610627	T/A	Missense variant	164	164	L/Q	Deleterious	0	Protein coding
16.	rs452194288	14	610638	610638	A/C	Missense variant	168	168	N/H	Deleterious	0	Protein coding
17.	rs137745035	14	610695	610695	A/G	Missense variant	187	187	T/A	Deleterious	0	Protein coding
18.	rs469935994	14	610791	610791	T/A	Missense variant	189	189	V/E	Deleterious	0	Protein coding
19.	rs452322000	14	610796	610796	T/A/G	Missense variant	191	191	S/T	Deleterious	0	Protein coding
20.	rs464323551	14	610797	610797	C/T	Missense variant	191	191	S/F	Deleterious	0	Protein coding
21.	rs459252534	14	610824	610824	T/C	Missense variant	200	200	I/T	Deleterious	0	Protein coding
22.	rs480898548	14	610826	610826	C/T	Missense variant	201	201	L/F	Deleterious	0	Protein coding
23.	rs445850328	14	610845	610845	C/T	Missense variant	207	207	S/F	Deleterious	0	Protein coding
24.	rs434545946	14	610848	610848	A/T	Missense variant	208	208	Y/F	Deleterious	0	Protein coding
25.	rs468699385	14	610857	610857	T/G	Missense variant	211	211	V/G	Deleterious	0	Protein coding
26.	rs457374450	14	610859	610859	A/C	Missense variant	212	212	N/H	Deleterious	0	Protein coding
27.	rs475760304	14	610860	610860	A/T	Missense variant	212	212	N/I	Deleterious	0	Protein coding
28.	rs451872523	14	610865	610865	T/C	Missense variant	214	214	W/R	Deleterious	0	Protein coding
29.	rs440590330	14	610868	610868	T/A/G	Missense variant	215	215	C/S	Deleterious	0	Protein coding
30.	rs440590330	14	610868	610868	T/A/G	Missense variant	215	215	C/G	Deleterious	0	Protein coding

31.	rs441665893	14	610880	610880	A/G	Missense variant	219	219	R/G	Deleterious	0	Protein coding
32.	rs467937945	14	611008	611008	T/G	Missense variant	228	228	L/W	Deleterious	0	Protein coding
33.	rs466703053	14	611050	611050	T/G	Missense variant	242	242	V/G	Deleterious	0	Protein coding
34.	rs473782024	14	611056	611056	A/C/G	Missense variant	244	244	Y/S	Deleterious	0	Protein coding
35.	rs473782024	14	611056	611056	A/C/G	Missense variant	244	244	Y/C	Deleterious	0	Protein coding
36.	rs456383648	14	611059	611059	C/G	Missense variant	245	245	P/R	Deleterious	0	Protein coding
37.	rs460163077	14	611070	611070	A/C	Missense variant	249	249	T/P	Deleterious	0	Protein coding
38.	rs466363475	14	611159	611159	A/C/G	Missense variant	255	255	Y/S	Deleterious	0	Protein coding
39.	rs466363475	14	611159	611159	A/C/G	Missense variant	255	255	Y/C	Deleterious	0	Protein coding
40.	rs433664097	14	611161	611161	T/G	Missense variant	256	256	F/V	Deleterious	0	Protein coding
41.	rs467358762	14	611165	611165	T/C	Missense variant	257	257	L/P	Deleterious	0	Protein coding
42.	rs465308299	14	611176	611176	A/C	Missense variant	261	261	T/P	Deleterious	0	Protein coding
43.	rs432244891	14	611177	611177	C/A	Missense variant	261	261	T/N	Deleterious	0	Protein coding
44.	rs453828909	14	611198	611198	T/G	Missense variant	268	268	F/C	Deleterious	0	Protein coding
45.	rs442892557	14	611203	611203	C/G	Missense variant	270	270	R/G	Deleterious	0	Protein coding
46.	rs454986719	14	611204	611204	G/C	Missense variant	270	270	R/P	Deleterious	0	Protein coding
47.	rs476704887	14	611206	611206	T/G	Missense variant	271	271	S/A	Deleterious	0	Protein coding
48.	rs443648310	14	611213	611213	G/T	Missense variant	273	273	R/L	Deleterious	0	Protein coding
49.	rs458871343	14	611221	611221	A/C	Missense variant	276	276	K/Q	Deleterious	0	Protein coding
50.	rs477786839	14	611222	611222	A/C	Missense variant	276	276	K/T	Deleterious	0	Protein coding

51.	rs481582054	14	611240	611240	G/T	Missense variant	282	282	R/L	Deleterious	0	Protein coding
52.	rs482774761	14	611243	611243	T/A/C	Missense variant	283	283	L/H	Deleterious	0	Protein coding
53.	rs482774761	14	611243	611243	T/A/C	Missense variant	283	283	L/P	Deleterious	0	Protein coding
54.	rs464924447	14	611252	611252	T/G	Missense variant	286	286	M/R	Deleterious	0	Protein coding
55.	rs452487622	14	611351	611351	T/C	Missense variant	289	289	L/P	Deleterious	0	Protein coding
56.	rs434800603	14	611357	611357	A/C	Missense variant	291	291	Q/P	Deleterious	0	Protein coding
57.	rs453311475	14	611360	611360	T/C	Missense variant	292	292	L/P	Deleterious	0	Protein coding
58.	rs475340943	14	611366	611366	T/G	Missense variant	294	294	V/G	Deleterious	0	Protein coding
59.	rs442291007	14	611371	611371	C/G	Missense variant	296	296	L/V	Deleterious	0	Protein coding
60.	rs134083952	14	611917	611917	T/C	Missense variant	374	374	F/L	Deleterious	0	Protein coding
61.	rs464356662	14	612238	612238	G/T	Missense variant	425	425	R/L	Deleterious	0	Protein coding
62.	rs433461918	14	612370	612370	T/A	Missense variant	445	445	V/E	Deleterious	0	Protein coding
63.	rs451795695	14	612393	612393	T/G	Missense variant	453	453	Y/D	Deleterious	0	Protein coding
64.	rs473363754	14	612394	612394	A/C	Missense variant	453	453	Y/S	Deleterious	0	Protein coding
65.	rs481100601	14	612405	612405	G/T	Missense variant	457	457	A/S	Deleterious	0	Protein coding
66.	rs474703848	14	612411	612411	T/G	Missense variant	459	459	W/G	Deleterious	0	Protein coding
67.	rs463356279	14	612421	612421	T/G	Missense variant	462	462	L/R	Deleterious	0	Protein coding
68.	rs135329220	14	612439	612439	T/G	Missense variant	468	468	V/G	Deleterious	0	Protein coding
69.	rs439482959	14	612442	612442	C/T	Missense variant	469	469	A/V	Deleterious	0	Protein coding
70.	rs457944656	14	612445	612445	T/G	Missense variant	470	470	V/G	Deleterious	0	Protein coding

71.	rs468682477	14	612466	612466	A/C/G	Missense variant	477	477	Y/S	Deleterious	0	Protein coding
72.	rs468682477	14	612466	612466	A/C/G	Missense variant	477	477	Y/C	Deleterious	0	Protein coding
73.	rs450840796	14	612472	612472	T/G	Missense variant	479	479	V/G	Deleterious	0	Protein coding

Supplementary Table S2. List of DGAT1 missense nsSNPs predicted to be deleterious by Protein Variation Effect Analyzer (PROVEAN) and other sequence-based tools. Bold indicate the selected variants for structural and conformational studies.

Wild residue	Position	Target residue	PROVEAN	PROVEAN score	PredictSNP prediction	PredictSNP expected accuracy	MAPP prediction	MAPP expected accuracy	PhD-SNP prediction	PhD-SNP expected accuracy	PolyPhen-1 prediction	PolyPhen-1 expected accuracy	PolyPhen-2 prediction	PolyPhen-2 expected accuracy	SNAP prediction	SNAP expected accuracy
R	12	G	NEUTRAL	-1.872	NEUTRAL	0.74796037	NEUTRAL	0.74449339	NEUTRAL	0.71871412	UNKNOWN	0	UNKNOWN	0	NEUTRAL	0.5
S	17	W	NEUTRAL	-2.174	NEUTRAL	0.6025641	NEUTRAL	0.65286344	NEUTRAL	0.58230958	UNKNOWN	0	UNKNOWN	0	DELETERIOUS	0.62
D	51	Y	NEUTRAL	-1.601	DELETERIOUS	0.54946365	NEUTRAL	0.6778169	NEUTRAL	0.58230958	DELETERIOUS	0.59445019	DELETERIOUS	0.3984576	DELETERIOUS	0.62
L	63	P	NEUTRAL	-1.624	NEUTRAL	0.73834499	NEUTRAL	0.63348018	NEUTRAL	0.50824588	NEUTRAL	0.66884082	NEUTRAL	0.6763527	NEUTRAL	0.71
H	66	R	DELETERIOUS	-7.014	NEUTRAL	0.6025641	NEUTRAL	0.72246696	DELETERIOUS	0.57795276	NEUTRAL	0.66884082	DELETERIOUS	0.5025707	NEUTRAL	0.71
D	70	Y	DELETERIOUS	-7.014	DELETERIOUS	0.60548272	NEUTRAL	0.72246696	DELETERIOUS	0.57795276	DELETERIOUS	0.74491225	DELETERIOUS	0.5623393	NEUTRAL	0.67
C	89	W	DELETERIOUS	-7.145	DELETERIOUS	0.86908365	DELETERIOUS	0.42653673	DELETERIOUS	0.88474971	DELETERIOUS	0.74491225	DELETERIOUS	0.6752412	DELETERIOUS	0.81
P	114	T	DELETERIOUS	-7.499	NEUTRAL	0.63151762	NEUTRAL	0.71742958	NEUTRAL	0.660879	NEUTRAL	0.66884082	DELETERIOUS	0.4312339	NEUTRAL	0.71
Y	126	C	DELETERIOUS	-5.815	NEUTRAL	0.73688811	NEUTRAL	0.76563877	DELETERIOUS	0.58885542	NEUTRAL	0.66884082	NEUTRAL	0.7077077	NEUTRAL	0.61
S	127	R	DELETERIOUS	-3.315	NEUTRAL	0.6025641	NEUTRAL	0.70484581	DELETERIOUS	0.67620995	NEUTRAL	0.66884082	NEUTRAL	0.7274549	DELETERIOUS	0.56
**W**	**128**	**R**	**DELETERIOUS**	**-9.113**	**DELETERIOUS**	**0.86908365**	**DELETERIOUS**	**0.78335832**	**DELETERIOUS**	**0.88474971**	**DELETERIOUS**	**0.74491225**	**DELETERIOUS**	**0.5623393**	**DELETERIOUS**	**0.81**
L	156	R	DELETERIOUS	-5.505	DELETERIOUS	0.60548272	NEUTRAL	0.72246696	DELETERIOUS	0.73260309	DELETERIOUS	0.74491225	DELETERIOUS	0.4530848	NEUTRAL	0.5
L	156	P	DELETERIOUS	-5.053	DELETERIOUS	0.60548272	DELETERIOUS	0.65667166	DELETERIOUS	0.85822785	NEUTRAL	0.66884082	NEUTRAL	0.7027027	DELETERIOUS	0.81
G	161	V	DELETERIOUS	-7.322	NEUTRAL	0.62831343	NEUTRAL	0.76563877	NEUTRAL	0.50824588	NEUTRAL	0.66884082	NEUTRAL	0.6436436	DELETERIOUS	0.62
L	164	Q	DELETERIOUS	-4.61	DELETERIOUS	0.7556615	DELETERIOUS	0.71814093	DELETERIOUS	0.85822785	DELETERIOUS	0.74491225	DELETERIOUS	0.5417738	NEUTRAL	0.5
N	168	H	DELETERIOUS	-4.475	DELETERIOUS	0.86908365	DELETERIOUS	0.76086957	DELETERIOUS	0.81731169	DELETERIOUS	0.74491225	DELETERIOUS	0.8114289	DELETERIOUS	0.62
T	187	A	DELETERIOUS	-2.866	NEUTRAL	0.71278765	NEUTRAL	0.65286344	NEUTRAL	0.7828765	NEUTRAL	0.66884082	DELETERIOUS	0.3984576	NEUTRAL	0.55
V	189	E	DELETERIOUS	-4.434	DELETERIOUS	0.78903456	DELETERIOUS	0.9137931	NEUTRAL	0.44670846	DELETERIOUS	0.74491225	DELETERIOUS	0.5623393	DELETERIOUS	0.72
S	191	F	NEUTRAL	-1.885	NEUTRAL	0.63151762	DELETERIOUS	0.58889722	NEUTRAL	0.44670846	NEUTRAL	0.66884082	NEUTRAL	0.6096096	NEUTRAL	0.61
S	191	T	DELETERIOUS	-3.937	NEUTRAL	0.65307311	DELETERIOUS	0.58889722	NEUTRAL	0.7828765	NEUTRAL	0.66884082	NEUTRAL	0.6936937	NEUTRAL	0.55
I	200	T	DELETERIOUS	-3.696	NEUTRAL	0.62831343	NEUTRAL	0.6969163	NEUTRAL	0.44670846	NEUTRAL	0.66884082	DELETERIOUS	0.4742931	NEUTRAL	0.55
L	201	F	DELETERIOUS	-2.863	NEUTRAL	0.73834499	NEUTRAL	0.78325991	NEUTRAL	0.83210379	NEUTRAL	0.66884082	NEUTRAL	0.7027027	NEUTRAL	0.61

S	207	F	DELETERIOUS	-5.77	DELETERIOUS	0.7556615	DELETERIOUS	0.7711928	DELETERIOUS	0.89325843	DELETERIOUS	0.74491225	DELETERIOUS	0.6497429	NEUTRAL	0.5
Y	208	F	DELETERIOUS	-3.513	NEUTRAL	0.6025641	DELETERIOUS	0.5089955	NEUTRAL	0.660879	NEUTRAL	0.66884082	DELETERIOUS	0.3984576	NEUTRAL	0.58
V	211	G	DELETERIOUS	-5.765	DELETERIOUS	0.71871275	NEUTRAL	0.70484581	DELETERIOUS	0.87523992	DELETERIOUS	0.74491225	DELETERIOUS	0.6008997	DELETERIOUS	0.81
N	212	H	DELETERIOUS	-4.808	DELETERIOUS	0.86908365	DELETERIOUS	0.76086957	DELETERIOUS	0.85822785	DELETERIOUS	0.74491225	DELETERIOUS	0.8114289	DELETERIOUS	0.72
N	212	I	DELETERIOUS	-8.655	DELETERIOUS	0.7556615	DELETERIOUS	0.40929535	DELETERIOUS	0.88474971	DELETERIOUS	0.74491225	DELETERIOUS	0.8114289	NEUTRAL	0.58
**W**	**214**	**R**	**DELETERIOUS**	**-13.43**	**DELETERIOUS**	**0.71871275**	**NEUTRAL**	**0.70484581**	**DELETERIOUS**	**0.73260309**	**DELETERIOUS**	**0.74491225**	**DELETERIOUS**	**0.5623393**	**DELETERIOUS**	**0.89**
C	215	S	DELETERIOUS	-8.363	DELETERIOUS	0.54946365	NEUTRAL	0.74977974	DELETERIOUS	0.85822785	NEUTRAL	0.66884082	DELETERIOUS	0.4742931	DELETERIOUS	0.56
**C**	**215**	**G**	**DELETERIOUS**	**-10.244**	**DELETERIOUS**	**0.71871275**	**NEUTRAL**	**0.63348018**	**DELETERIOUS**	**0.87523992**	**DELETERIOUS**	**0.74491225**	**DELETERIOUS**	**0.5623393**	**DELETERIOUS**	**0.62**
R	219	G	DELETERIOUS	-4.439	DELETERIOUS	0.71871275	NEUTRAL	0.74449339	DELETERIOUS	0.7733853	DELETERIOUS	0.74491225	DELETERIOUS	0.4742931	DELETERIOUS	0.72
L	228	W	NEUTRAL	-1.411	NEUTRAL	0.6025641	NEUTRAL	0.65022026	NEUTRAL	0.55202703	DELETERIOUS	0.74491225	DELETERIOUS	0.4312339	NEUTRAL	0.77
V	242	G	DELETERIOUS	-5.412	DELETERIOUS	0.65494636	NEUTRAL	0.74185022	DELETERIOUS	0.67620995	DELETERIOUS	0.59445019	DELETERIOUS	0.3984576	DELETERIOUS	0.56
Y	244	S	DELETERIOUS	-8.195	DELETERIOUS	0.86908365	DELETERIOUS	0.58770615	DELETERIOUS	0.88474971	DELETERIOUS	0.59445019	DELETERIOUS	0.5931877	DELETERIOUS	0.62
Y	244	C	DELETERIOUS	-8.409	DELETERIOUS	0.86908365	DELETERIOUS	0.48350825	DELETERIOUS	0.88474971	DELETERIOUS	0.74491225	DELETERIOUS	0.6008997	DELETERIOUS	0.72
**P**	**245**	**R**	**DELETERIOUS**	**-8.543**	**DELETERIOUS**	**0.86908365**	**DELETERIOUS**	**0.87706147**	**DELETERIOUS**	**0.87523992**	**DELETERIOUS**	**0.74491225**	**DELETERIOUS**	**0.8114289**	**DELETERIOUS**	**0.81**
T	249	P	DELETERIOUS	-4.513	DELETERIOUS	0.86908365	DELETERIOUS	0.65667166	DELETERIOUS	0.88474971	DELETERIOUS	0.74491225	DELETERIOUS	0.6752412	DELETERIOUS	0.72
Y	255	S	DELETERIOUS	-8.059	DELETERIOUS	0.65494636	NEUTRAL	0.73480176	DELETERIOUS	0.81731169	DELETERIOUS	0.59445019	DELETERIOUS	0.6343188	DELETERIOUS	0.81
Y	255	C	DELETERIOUS	-8.226	DELETERIOUS	0.7556615	NEUTRAL	0.6778169	DELETERIOUS	0.88474971	DELETERIOUS	0.74491225	DELETERIOUS	0.8114289	DELETERIOUS	0.85
F	256	V	DELETERIOUS	-6.698	DELETERIOUS	0.86908365	DELETERIOUS	0.78335832	DELETERIOUS	0.87523992	DELETERIOUS	0.74491225	DELETERIOUS	0.8114289	DELETERIOUS	0.85
L	257	P	DELETERIOUS	-4.985	DELETERIOUS	0.86908365	DELETERIOUS	0.85832084	DELETERIOUS	0.81731169	DELETERIOUS	0.74491225	DELETERIOUS	0.6343188	DELETERIOUS	0.85
T	261	N	DELETERIOUS	-5.795	DELETERIOUS	0.86908365	DELETERIOUS	0.8065967	DELETERIOUS	0.88474971	DELETERIOUS	0.74491225	DELETERIOUS	0.8114289	DELETERIOUS	0.81
T	261	P	DELETERIOUS	-4.829	DELETERIOUS	0.86908365	DELETERIOUS	0.9137931	DELETERIOUS	0.88474971	DELETERIOUS	0.74491225	DELETERIOUS	0.8114289	DELETERIOUS	0.72
F	268	C	DELETERIOUS	-7.707	DELETERIOUS	0.86908365	DELETERIOUS	0.78335832	DELETERIOUS	0.87523992	DELETERIOUS	0.74491225	DELETERIOUS	0.6752412	DELETERIOUS	0.72
R	270	G	DELETERIOUS	-6.586	DELETERIOUS	0.86908365	DELETERIOUS	0.76611694	DELETERIOUS	0.88474971	DELETERIOUS	0.74491225	DELETERIOUS	0.6752412	DELETERIOUS	0.62
R	270	P	DELETERIOUS	-6.528	DELETERIOUS	0.86908365	DELETERIOUS	0.7711928	DELETERIOUS	0.88474971	DELETERIOUS	0.74491225	DELETERIOUS	0.8114289	DELETERIOUS	0.81
S	271	A	NEUTRAL	-1.894	NEUTRAL	0.63151762	DELETERIOUS	0.58770615	NEUTRAL	0.50824588	NEUTRAL	0.66884082	NEUTRAL	0.7024048	NEUTRAL	0.5
R	273	L	DELETERIOUS	-5.761	DELETERIOUS	0.60548272	NEUTRAL	0.64229075	DELETERIOUS	0.60798122	DELETERIOUS	0.59445019	DELETERIOUS	0.5931877	NEUTRAL	0.5
K	276	Q	DELETERIOUS	-3.28	DELETERIOUS	0.54946365	NEUTRAL	0.77004405	DELETERIOUS	0.58885542	DELETERIOUS	0.59445019	DELETERIOUS	0.5417738	NEUTRAL	0.55

K	276	T	DELETERIOUS	-5.062	NEUTRAL	0.62831343	NEUTRAL	0.74977974	NEUTRAL	0.50824588	NEUTRAL	0.66884082	DELETERIOUS	0.4530848	NEUTRAL	0.5
R	282	L	DELETERIOUS	-6.761	DELETERIOUS	0.50595948	NEUTRAL	0.6778169	DELETERIOUS	0.60798122	NEUTRAL	0.66884082	DELETERIOUS	0.5623393	NEUTRAL	0.5
L	283	H	DELETERIOUS	-5.266	DELETERIOUS	0.7556615	DELETERIOUS	0.76611694	NEUTRAL	0.58230958	DELETERIOUS	0.74491225	DELETERIOUS	0.5507712	DELETERIOUS	0.62
L	283	P	DELETERIOUS	-5.104	DELETERIOUS	0.86908365	DELETERIOUS	0.65667166	DELETERIOUS	0.7733853	DELETERIOUS	0.74491225	DELETERIOUS	0.6497429	DELETERIOUS	0.72
M	286	R	DELETERIOUS	-4.692	DELETERIOUS	0.7556615	DELETERIOUS	0.9137931	NEUTRAL	0.55202703	DELETERIOUS	0.59445019	DELETERIOUS	0.6008997	DELETERIOUS	0.72
L	289	P	DELETERIOUS	-3.753	DELETERIOUS	0.86908365	DELETERIOUS	0.58770615	DELETERIOUS	0.67620995	DELETERIOUS	0.74491225	DELETERIOUS	0.6008997	DELETERIOUS	0.62
Q	291	P	DELETERIOUS	-4.373	DELETERIOUS	0.65494636	DELETERIOUS	0.55997001	DELETERIOUS	0.60798122	DELETERIOUS	0.59445019	DELETERIOUS	0.5931877	DELETERIOUS	0.62
L	292	P	DELETERIOUS	-5.925	DELETERIOUS	0.86908365	DELETERIOUS	0.78335832	DELETERIOUS	0.67620995	DELETERIOUS	0.74491225	DELETERIOUS	0.8114289	DELETERIOUS	0.85
V	294	G	DELETERIOUS	-5.147	NEUTRAL	0.73834499	NEUTRAL	0.65903084	NEUTRAL	0.660879	NEUTRAL	0.66884082	NEUTRAL	0.6766767	NEUTRAL	0.77
L	296	V	NEUTRAL	-2.393	NEUTRAL	0.68365861	NEUTRAL	0.6778169	NEUTRAL	0.68183996	NEUTRAL	0.66884082	DELETERIOUS	0.4312339	NEUTRAL	0.67
F	374	L	DELETERIOUS	-5.37	DELETERIOUS	0.86908365	DELETERIOUS	0.71814093	DELETERIOUS	0.88474971	DELETERIOUS	0.74491225	DELETERIOUS	0.8114289	DELETERIOUS	0.62
R	425	L	DELETERIOUS	-4.56	DELETERIOUS	0.50595948	DELETERIOUS	0.76086957	DELETERIOUS	0.88474971	NEUTRAL	0.66884082	NEUTRAL	0.6276276	NEUTRAL	0.5
V	445	E	DELETERIOUS	-3.352	DELETERIOUS	0.60548272	DELETERIOUS	0.84257871	DELETERIOUS	0.67620995	NEUTRAL	0.66884082	DELETERIOUS	0.4530848	NEUTRAL	0.55
Y	453	S	DELETERIOUS	-6.275	NEUTRAL	0.63151762	NEUTRAL	0.65903084	DELETERIOUS	0.67620995	NEUTRAL	0.66884082	DELETERIOUS	0.4530848	NEUTRAL	0.5
Y	453	D	DELETERIOUS	-5.233	DELETERIOUS	0.86908365	DELETERIOUS	0.9137931	DELETERIOUS	0.81731169	DELETERIOUS	0.59445019	DELETERIOUS	0.6343188	DELETERIOUS	0.62
A	457	S	NEUTRAL	-1.863	DELETERIOUS	0.52145411	DELETERIOUS	0.5089955	NEUTRAL	0.58230958	NEUTRAL	0.66884082	DELETERIOUS	0.4742931	DELETERIOUS	0.56
**W**	**459**	**G**	**DELETERIOUS**	**-11.385**	**DELETERIOUS**	**0.86908365**	**DELETERIOUS**	**0.81934033**	**DELETERIOUS**	**0.88474971**	**DELETERIOUS**	**0.74491225**	**DELETERIOUS**	**0.8114289**	**DELETERIOUS**	**0.87**
L	462	R	DELETERIOUS	-4.971	DELETERIOUS	0.86908365	DELETERIOUS	0.85682159	DELETERIOUS	0.87523992	DELETERIOUS	0.74491225	DELETERIOUS	0.6752412	DELETERIOUS	0.85
V	468	G	DELETERIOUS	-4.889	DELETERIOUS	0.54946365	DELETERIOUS	0.46176912	DELETERIOUS	0.81731169	NEUTRAL	0.66884082	DELETERIOUS	0	NEUTRAL	0.55
A	469	V	DELETERIOUS	-2.834	NEUTRAL	0.65307311	NEUTRAL	0.71742958	DELETERIOUS	0.57795276	NEUTRAL	0.66884082	NEUTRAL	0.7077077	NEUTRAL	0.55
V	470	G	DELETERIOUS	-6.136	DELETERIOUS	0.50595948	NEUTRAL	0.65903084	DELETERIOUS	0.81731169	NEUTRAL	0.66884082	DELETERIOUS	0.4312339	NEUTRAL	0.61
Y	477	S	DELETERIOUS	-7.092	DELETERIOUS	0.60697259	NEUTRAL	0.64229075	DELETERIOUS	0.88474971	DELETERIOUS	0.74491225	DELETERIOUS	0.8114289	NEUTRAL	0.58
Y	477	C	DELETERIOUS	-7.025	DELETERIOUS	0.7556615	NEUTRAL	0.65022026	DELETERIOUS	0.7733853	DELETERIOUS	0.74491225	DELETERIOUS	0.8114289	DELETERIOUS	0.56
V	479	G	DELETERIOUS	-5.711	DELETERIOUS	0.71871275	NEUTRAL	0.6778169	DELETERIOUS	0.7733853	DELETERIOUS	0.74491225	DELETERIOUS	0.5623393	DELETERIOUS	0.62

Supplementary Table S3. The interacting partners of the DGAT1 with their respective scores predicted by STRING database.

node1	node2	node1_string_id	node2_string_id	neighborhood_on_chromosome	gene_fusion	phylogenetic_cooccurrence	homology	coexpression	experimentally_determined_interaction	database_annotated	automated_textmining	combined_score
ADH4	AWAT2	9913.ENSBTAP00000003769	9913.ENSBTAP00000016283	0	0	0	0	0.1	0	0.9	0.045	0.901
ADH4	DGAT1	9913.ENSBTAP00000003769	9913.ENSBTAP00000037256	0	0	0	0	0	0	0.9	0.095	0.905
ADH4	CYP27C1	9913.ENSBTAP00000003769	9913.ENSBTAP00000052365	0	0	0	0	0.1	0	0.91	0	0.91
ADH4	RETSAT	9913.ENSBTAP00000003769	9913.ENSBTAP00000029241	0	0	0	0	0.1	0	0.92	0.16	0.929
ADH4	CYP26A1	9913.ENSBTAP00000003769	9913.ENSBTAP00000028140	0	0	0	0	0.1	0	0.91	0.252	0.932
ADH5	AWAT2	9913.ENSBTAP00000021304	9913.ENSBTAP00000016283	0	0	0	0	0.1	0	0.9	0.045	0.901
ADH5	CYP27C1	9913.ENSBTAP00000021304	9913.ENSBTAP00000052365	0	0	0	0	0	0	0.91	0	0.908
ADH5	DGAT1	9913.ENSBTAP00000021304	9913.ENSBTAP00000037256	0	0	0	0	0	0	0.9	0.134	0.909
ADH5	CYP26A1	9913.ENSBTAP00000021304	9913.ENSBTAP00000028140	0	0	0	0	0.1	0	0.91	0.332	0.939
ADH5	RETSAT	9913.ENSBTAP00000021304	9913.ENSBTAP00000029241	0	0	0	0	0.1	0	0.92	0.366	0.946
ADH6	AWAT2	9913.ENSBTAP00000009191	9913.ENSBTAP00000016283	0	0	0	0	0.1	0	0.9	0.045	0.901
ADH6	DGAT1	9913.ENSBTAP00000009191	9913.ENSBTAP00000037256	0	0	0	0	0	0	0.9	0.071	0.903
ADH6	CYP27C1	9913.ENSBTAP00000009191	9913.ENSBTAP00000052365	0	0	0	0	0	0	0.91	0.056	0.909
ADH6	RETSAT	9913.ENSBTAP00000009191	9913.ENSBTAP00000029241	0	0	0	0	0.1	0	0.92	0	0.919
ADH6	CYP26A1	9913.ENSBTAP00000009191	9913.ENSBTAP00000028140	0	0	0	0	0.1	0	0.91	0.179	0.925
ADH7	AWAT2	9913.ENSBTAP00000020879	9913.ENSBTAP00000016283	0	0	0	0	0.1	0	0.9	0.045	0.901
ADH7	DGAT1	9913.ENSBTAP00000020879	9913.ENSBTAP00000037256	0	0	0	0	0	0	0.9	0.061	0.902
ADH7	CYP27C1	9913.ENSBTAP00000020879	9913.ENSBTAP00000052365	0	0	0	0	0.1	0	0.91	0.059	0.911
ADH7	CYP26A1	9913.ENSBTAP00000020879	9913.ENSBTAP00000028140	0	0	0	0	0.1	0	0.91	0.168	0.924
ADH7	RETSAT	9913.ENSBTAP00000020879	9913.ENSBTAP00000029241	0	0	0	0	0.1	0	0.92	0.104	0.924
AWAT2	RDH11	9913.ENSBTAP00000016283	9913.ENSBTAP00000002535	0	0	0	0	0.1	0	0.9	0.049	0.903
AWAT2	ADH4	9913.ENSBTAP00000016283	9913.ENSBTAP00000003769	0	0	0	0	0.1	0	0.9	0.045	0.901
AWAT2	ADH6	9913.ENSBTAP00000016283	9913.ENSBTAP00000009191	0	0	0	0	0.1	0	0.9	0.045	0.901
AWAT2	HSD17B6	9913.ENSBTAP00000016283	9913.ENSBTAP00000015564	0	0	0	0	0	0	0.9	0	0.9
AWAT2	RDH5	9913.ENSBTAP00000016283	9913.ENSBTAP00000056512	0	0	0	0	0	0	0.9	0	0.9

AWAT2	CYP27C1	9913.ENSBTAP00000016283	9913.ENSBTAP00000052365	0	0	0	0	0	0	0.9	0	0.9
AWAT2	DHRS9	9913.ENSBTAP00000016283	9913.ENSBTAP00000055524	0	0	0	0	0	0	0.9	0	0.9
AWAT2	ADH7	9913.ENSBTAP00000016283	9913.ENSBTAP00000020879	0	0	0	0	0.1	0	0.9	0.045	0.901
AWAT2	RPE65	9913.ENSBTAP00000016283	9913.ENSBTAP00000041254	0	0	0	0	0	0	0.9	0.051	0.901
AWAT2	ADH5	9913.ENSBTAP00000016283	9913.ENSBTAP00000021304	0	0	0	0	0.1	0	0.9	0.045	0.901
AWAT2	DGAT1	9913.ENSBTAP00000016283	9913.ENSBTAP00000037256	0	0	0	0	0.1	0	0.8	0.406	0.901
AWAT2	CYP26A1	9913.ENSBTAP00000016283	9913.ENSBTAP00000028140	0	0	0	0	0.1	0	0.9	0.043	0.902
AWAT2	DHRS4	9913.ENSBTAP00000016283	9913.ENSBTAP00000023493	0	0	0	0	0.1	0	0.9	0.049	0.902
AWAT2	RDH10	9913.ENSBTAP00000016283	9913.ENSBTAP00000026830	0	0	0	0	0.1	0	0.9	0.074	0.904
AWAT2	RETSAT	9913.ENSBTAP00000016283	9913.ENSBTAP00000029241	0	0	0	0	0.1	0	0.9	0.069	0.904
AWAT2	DHRS3	9913.ENSBTAP00000016283	9913.ENSBTAP00000033930	0	0	0	0	0.1	0	0.9	0.074	0.904
AWAT2	SDR16C5	9913.ENSBTAP00000016283	9913.ENSBTAP00000024714	0	0	0	0	0.1	0	0.9	0.074	0.904
CYP26A1	RDH11	9913.ENSBTAP00000028140	9913.ENSBTAP00000002535	0	0	0	0	0	0	0.91	0.378	0.94
CYP26A1	ADH4	9913.ENSBTAP00000028140	9913.ENSBTAP00000003769	0	0	0	0	0.1	0	0.91	0.252	0.932
CYP26A1	ADH6	9913.ENSBTAP00000028140	9913.ENSBTAP00000009191	0	0	0	0	0.1	0	0.91	0.179	0.925
CYP26A1	HSD17B6	9913.ENSBTAP00000028140	9913.ENSBTAP00000015564	0	0	0	0	0.1	0	0.9	0.068	0.907
CYP26A1	AWAT2	9913.ENSBTAP00000028140	9913.ENSBTAP00000016283	0	0	0	0	0.1	0	0.9	0.043	0.902
CYP26A1	ADH7	9913.ENSBTAP00000028140	9913.ENSBTAP00000020879	0	0	0	0	0.1	0	0.91	0.168	0.924
CYP26A1	ADH5	9913.ENSBTAP00000028140	9913.ENSBTAP00000021304	0	0	0	0	0.1	0	0.91	0.332	0.939
CYP26A1	DHRS4	9913.ENSBTAP00000028140	9913.ENSBTAP00000023493	0	0	0	0	0.1	0	0.91	0.108	0.916
CYP26A1	SDR16C5	9913.ENSBTAP00000028140	9913.ENSBTAP00000024714	0	0	0	0	0	0	0.91	0.366	0.941
CYP26A1	RDH10	9913.ENSBTAP00000028140	9913.ENSBTAP00000026830	0	0	0	0	0	0	0.91	0.765	0.978
CYP26A1	DGAT1	9913.ENSBTAP00000028140	9913.ENSBTAP00000037256	0	0	0	0	0.1	0	0.9	0.14	0.913
CYP26A1	RETSAT	9913.ENSBTAP00000028140	9913.ENSBTAP00000029241	0	0	0	0	0.1	0	0.9	0.248	0.923
CYP26A1	CYP27C1	9913.ENSBTAP00000028140	9913.ENSBTAP00000052365	0	0	0	1	0.1	0	0.9	0.385	0.935
CYP26A1	DHRS9	9913.ENSBTAP00000028140	9913.ENSBTAP00000055524	0	0	0	0	0	0	0.9	0.539	0.953
CYP26A1	DHRS3	9913.ENSBTAP00000028140	9913.ENSBTAP00000033930	0	0	0	0	0.1	0	0.91	0.72	0.974
CYP27C1	RDH11	9913.ENSBTAP00000052365	9913.ENSBTAP00000002535	0	0	0	0	0	0	0.91	0.068	0.91
CYP27C1	ADH4	9913.ENSBTAP00000052365	9913.ENSBTAP00000003769	0	0	0	0	0.1	0	0.91	0	0.91

CYP27C1	ADH6	9913.ENSBTAP00000052365	9913.ENSBTAP00000009191	0	0	0	0	0	0	0.91	0.056	0.909
CYP27C1	HSD17B6	9913.ENSBTAP00000052365	9913.ENSBTAP00000015564	0	0	0	0	0.1	0	0.9	0.043	0.903
CYP27C1	AWAT2	9913.ENSBTAP00000052365	9913.ENSBTAP00000016283	0	0	0	0	0	0	0.9	0	0.9
CYP27C1	ADH7	9913.ENSBTAP00000052365	9913.ENSBTAP00000020879	0	0	0	0	0.1	0	0.91	0.059	0.911
CYP27C1	ADH5	9913.ENSBTAP00000052365	9913.ENSBTAP00000021304	0	0	0	0	0	0	0.91	0	0.908
CYP27C1	DHRS4	9913.ENSBTAP00000052365	9913.ENSBTAP00000023493	0	0	0	0	0	0	0.91	0.057	0.909
CYP27C1	SDR16C5	9913.ENSBTAP00000052365	9913.ENSBTAP00000024714	0	0	0	0	0	0	0.91	0.043	0.908
CYP27C1	RDH10	9913.ENSBTAP00000052365	9913.ENSBTAP00000026830	0	0	0	0	0	0	0.91	0.043	0.908
CYP27C1	CYP26A1	9913.ENSBTAP00000052365	9913.ENSBTAP00000028140	0	0	0	1	0.1	0	0.9	0.385	0.935
CYP27C1	RETSAT	9913.ENSBTAP00000052365	9913.ENSBTAP00000029241	0	0	0	0	0.1	0	0.9	0.307	0.929
CYP27C1	DHRS3	9913.ENSBTAP00000052365	9913.ENSBTAP00000033930	0	0	0	0	0	0	0.91	0.054	0.909
CYP27C1	DGAT1	9913.ENSBTAP00000052365	9913.ENSBTAP00000037256	0	0	0	0	0.1	0	0.9	0.06	0.904
CYP27C1	DHRS9	9913.ENSBTAP00000052365	9913.ENSBTAP00000055524	0	0	0	0	0	0	0.9	0	0.901
DGAT1	PPAP2C	9913.ENSBTAP00000037256	9913.ENSBTAP00000000955	0	0	0	0	0	0	0.99	0.177	0.995
DGAT1	DGAT2	9913.ENSBTAP00000037256	9913.ENSBTAP00000001536	0	0	0	0	0.1	0	0.8	0.917	0.988
DGAT1	RDH11	9913.ENSBTAP00000037256	9913.ENSBTAP00000002535	0	0	0	0	0	0	0.9	0.101	0.906
DGAT1	ADH4	9913.ENSBTAP00000037256	9913.ENSBTAP00000003769	0	0	0	0	0	0	0.9	0.095	0.905
DGAT1	LIPF	9913.ENSBTAP00000037256	9913.ENSBTAP00000008453	0	0	0	0	0.1	0	0.93	0.329	0.949
DGAT1	ADH6	9913.ENSBTAP00000037256	9913.ENSBTAP00000009191	0	0	0	0	0	0	0.9	0.071	0.903
DGAT1	MOGAT1	9913.ENSBTAP00000037256	9913.ENSBTAP00000011091	0	0	0	0	0.1	0	0.65	0.83	0.956
DGAT1	LPIN3	9913.ENSBTAP00000037256	9913.ENSBTAP00000012081	0	0	0	0	0.1	0	0.75	0.599	0.904
DGAT1	PPAP2B	9913.ENSBTAP00000037256	9913.ENSBTAP00000015460	0	0	0	0	0	0	0.99	0.145	0.994
DGAT1	HSD17B6	9913.ENSBTAP00000037256	9913.ENSBTAP00000015564	0	0	0	0	0.1	0	0.9	0.084	0.906
DGAT1	LPIN2	9913.ENSBTAP00000037256	9913.ENSBTAP00000015997	0	0	0	0	0.1	0	0.75	0.664	0.916
DGAT1	AWAT2	9913.ENSBTAP00000037256	9913.ENSBTAP00000016283	0	0	0	0	0.1	0	0.8	0.406	0.901
DGAT1	ADH7	9913.ENSBTAP00000037256	9913.ENSBTAP00000020879	0	0	0	0	0	0	0.9	0.061	0.902
DGAT1	ADH5	9913.ENSBTAP00000037256	9913.ENSBTAP00000021304	0	0	0	0	0	0	0.9	0.134	0.909
DGAT1	DHRS4	9913.ENSBTAP00000037256	9913.ENSBTAP00000023493	0	0	0	0	0.1	0	0.9	0.269	0.926
DGAT1	SDR16C5	9913.ENSBTAP00000037256	9913.ENSBTAP00000024714	0	0	0	0	0.1	0	0.9	0.388	0.937

DGAT1	LOC513388	9913.ENSBTAP00000037256	9913.ENSBTAP00000025329	0	0	0	0	0	0	0.95	0.145	0.95
DGAT1	RDH10	9913.ENSBTAP00000037256	9913.ENSBTAP00000026830	0	0	0	0	0	0	0.9	0.226	0.919
DGAT1	LOC618076	9913.ENSBTAP00000037256	9913.ENSBTAP00000026848	0	0	0	0	0.1	0	0.9	0.458	0.954
DGAT1	CYP26A1	9913.ENSBTAP00000037256	9913.ENSBTAP00000028140	0	0	0	0	0.1	0	0.9	0.14	0.913
DGAT1	RETSAT	9913.ENSBTAP00000037256	9913.ENSBTAP00000029241	0	0	0	0	0.1	0	0.9	0.146	0.912
DGAT1	PNPLA2	9913.ENSBTAP00000037256	9913.ENSBTAP00000029866	0	0	0	0	0.1	0	0.8	0.813	0.963
DGAT1	DHRS3	9913.ENSBTAP00000037256	9913.ENSBTAP00000033930	0	0	0	0	0	0	0.9	0.143	0.91
DGAT1	CYP27C1	9913.ENSBTAP00000037256	9913.ENSBTAP00000052365	0	0	0	0	0.1	0	0.9	0.06	0.904
DGAT1	RDH5	9913.ENSBTAP00000037256	9913.ENSBTAP00000056512	0	0	0	0	0.1	0	0.9	0.119	0.91
DGAT1	DHRS9	9913.ENSBTAP00000037256	9913.ENSBTAP00000055524	0	0	0	0	0.1	0	0.9	0.162	0.914
DGAT1	RPE65	9913.ENSBTAP00000037256	9913.ENSBTAP00000041254	0	0	0	0	0.1	0	0.9	0.354	0.936
DGAT1	LPPR2	9913.ENSBTAP00000037256	9913.ENSBTAP00000043142	0	0	0	0	0	0	0.95	0.145	0.95
DGAT1	LOC785379	9913.ENSBTAP00000037256	9913.ENSBTAP00000056386	0	0	0	0	0.1	0	0.9	0.345	0.952
DGAT1	LPIN1	9913.ENSBTAP00000037256	9913.ENSBTAP00000040997	0	0	0	0	0.1	0	0.75	0.841	0.96
DGAT1	ENSBTAG00000037483	9913.ENSBTAP00000037256	9913.ENSBTAP00000049702	0	0	0	0	0.1	0	0.9	0.597	0.97
DGAT1	PPAPDC1A	9913.ENSBTAP00000037256	9913.ENSBTAP00000042923	0	0	0	0	0	0	0.98	0.328	0.988
DGAT1	PPAPDC1B	9913.ENSBTAP00000037256	9913.ENSBTAP00000039585	0	0	0	0	0.1	0	0.98	0.328	0.989
DGAT1	PPAP2A	9913.ENSBTAP00000037256	9913.ENSBTAP00000044571	0	0	0	0	0	0	0.99	0.349	0.996
DGAT2	PPAP2C	9913.ENSBTAP00000001536	9913.ENSBTAP00000000955	0	0	0	0	0.1	0	0.99	0.56	0.997
DGAT2	LOC618076	9913.ENSBTAP00000001536	9913.ENSBTAP00000026848	0	0	0	1	0	0	0.9	0.531	0.902
DGAT2	LOC785379	9913.ENSBTAP00000001536	9913.ENSBTAP00000056386	0	0	0	1	0	0	0.9	0.328	0.91
DGAT2	ENSBTAG00000037483	9913.ENSBTAP00000001536	9913.ENSBTAP00000049702	0	0	0	1	0	0	0.9	0.768	0.912
DGAT2	LPIN2	9913.ENSBTAP00000001536	9913.ENSBTAP00000015997	0	0	0	0	0.1	0	0.73	0.721	0.925
DGAT2	LPIN1	9913.ENSBTAP00000001536	9913.ENSBTAP00000040997	0	0	0	0	0.1	0	0.73	0.78	0.941
DGAT2	LOC513388	9913.ENSBTAP00000001536	9913.ENSBTAP00000025329	0	0	0	0	0	0	0.95	0.083	0.947
DGAT2	LPPR2	9913.ENSBTAP00000001536	9913.ENSBTAP00000043142	0	0	0	0	0	0	0.95	0.083	0.947
DGAT2	PNPLA2	9913.ENSBTAP00000001536	9913.ENSBTAP00000029866	0	0	0	0	0.1	0	0.8	0.826	0.965
DGAT2	PPAPDC1A	9913.ENSBTAP00000001536	9913.ENSBTAP00000042923	0	0	0	0	0.1	0	0.98	0.199	0.986
DGAT2	PPAPDC1B	9913.ENSBTAP00000001536	9913.ENSBTAP00000039585	0	0	0	0	0.1	0	0.98	0.306	0.988

DGAT2	DGAT1	9913.ENSBTAP00000001536	9913.ENSBTAP00000037256	0	0	0	0	0.1	0	0.8	0.917	0.988
DGAT2	PPAP2B	9913.ENSBTAP00000001536	9913.ENSBTAP00000015460	0	0	0	0	0	0	0.99	0.397	0.996
DGAT2	PPAP2A	9913.ENSBTAP00000001536	9913.ENSBTAP00000044571	0	0	0	0	0	0	0.99	0.383	0.996
DHRS3	AWAT2	9913.ENSBTAP00000033930	9913.ENSBTAP00000016283	0	0	0	0	0.1	0	0.9	0.074	0.904
DHRS3	CYP26A1	9913.ENSBTAP00000033930	9913.ENSBTAP00000028140	0	0	0	0	0.1	0	0.91	0.72	0.974
DHRS3	RETSAT	9913.ENSBTAP00000033930	9913.ENSBTAP00000029241	0.1	0	0	0	0.1	0	0.9	0.373	0.938
DHRS3	CYP27C1	9913.ENSBTAP00000033930	9913.ENSBTAP00000052365	0	0	0	0	0	0	0.91	0.054	0.909
DHRS3	DGAT1	9913.ENSBTAP00000033930	9913.ENSBTAP00000037256	0	0	0	0	0	0	0.9	0.143	0.91
DHRS3	DHRS9	9913.ENSBTAP00000033930	9913.ENSBTAP00000055524	0	0	0	0	0	0	0.8	0.581	0.929
DHRS4	AWAT2	9913.ENSBTAP00000023493	9913.ENSBTAP00000016283	0	0	0	0	0.1	0	0.9	0.049	0.902
DHRS4	DHRS9	9913.ENSBTAP00000023493	9913.ENSBTAP00000055524	0	0	0	0	0	0	0.8	0.422	0.902
DHRS4	CYP27C1	9913.ENSBTAP00000023493	9913.ENSBTAP00000052365	0	0	0	0	0	0	0.91	0.057	0.909
DHRS4	CYP26A1	9913.ENSBTAP00000023493	9913.ENSBTAP00000028140	0	0	0	0	0.1	0	0.91	0.108	0.916
DHRS4	DGAT1	9913.ENSBTAP00000023493	9913.ENSBTAP00000037256	0	0	0	0	0.1	0	0.9	0.269	0.926
DHRS4	RETSAT	9913.ENSBTAP00000023493	9913.ENSBTAP00000029241	0.1	0	0	0	0.1	0	0.9	0.422	0.944
DHRS9	AWAT2	9913.ENSBTAP00000055524	9913.ENSBTAP00000016283	0	0	0	0	0	0	0.9	0	0.9
DHRS9	DHRS4	9913.ENSBTAP00000055524	9913.ENSBTAP00000023493	0	0	0	0	0	0	0.8	0.422	0.902
DHRS9	CYP26A1	9913.ENSBTAP00000055524	9913.ENSBTAP00000028140	0	0	0	0	0	0	0.9	0.539	0.953
DHRS9	RETSAT	9913.ENSBTAP00000055524	9913.ENSBTAP00000029241	0	0	0	0	0.1	0	0.9	0	0.901
DHRS9	DHRS3	9913.ENSBTAP00000055524	9913.ENSBTAP00000033930	0	0	0	0	0	0	0.8	0.581	0.929
DHRS9	DGAT1	9913.ENSBTAP00000055524	9913.ENSBTAP00000037256	0	0	0	0	0.1	0	0.9	0.162	0.914
DHRS9	CYP27C1	9913.ENSBTAP00000055524	9913.ENSBTAP00000052365	0	0	0	0	0	0	0.9	0	0.901
ENSBTAG00000037483	PPAP2C	9913.ENSBTAP00000049702	9913.ENSBTAP00000000955	0	0	0	0	0.1	0	0.86	0.396	0.915
ENSBTAG00000037483	DGAT2	9913.ENSBTAP00000049702	9913.ENSBTAP00000001536	0	0	0	1	0	0	0.9	0.768	0.912
ENSBTAG00000037483	DGAT1	9913.ENSBTAP00000049702	9913.ENSBTAP00000037256	0	0	0	0	0.1	0	0.9	0.597	0.97
ENSBTAG00000037483	PPAPDC1B	9913.ENSBTAP00000049702	9913.ENSBTAP00000039585	0	0	0	0	0.1	0	0.94	0.199	0.949
ENSBTAG00000037483	PPAPDC1A	9913.ENSBTAP00000049702	9913.ENSBTAP00000042923	0	0	0	0	0.1	0	0.94	0.199	0.949
HSD17B6	AWAT2	9913.ENSBTAP00000015564	9913.ENSBTAP00000016283	0	0	0	0	0	0	0.9	0	0.9
HSD17B6	CYP27C1	9913.ENSBTAP00000015564	9913.ENSBTAP00000052365	0	0	0	0	0.1	0	0.9	0.043	0.903

HSD17B6	DGAT1	9913.ENSBTAP00000015564	9913.ENSBTAP00000037256	0	0	0	0	0.1	0	0.9	0.084	0.906
HSD17B6	CYP26A1	9913.ENSBTAP00000015564	9913.ENSBTAP00000028140	0	0	0	0	0.1	0	0.9	0.068	0.907
HSD17B6	RETSAT	9913.ENSBTAP00000015564	9913.ENSBTAP00000029241	0	0	0	0	0.1	0	0.9	0.158	0.914
LIPF	DGAT1	9913.ENSBTAP00000008453	9913.ENSBTAP00000037256	0	0	0	0	0.1	0	0.93	0.329	0.949
LOC513388	DGAT2	9913.ENSBTAP00000025329	9913.ENSBTAP00000001536	0	0	0	0	0	0	0.95	0.083	0.947
LOC513388	MOGAT1	9913.ENSBTAP00000025329	9913.ENSBTAP00000011091	0	0	0	0	0	0	0.9	0.083	0.908
LOC513388	DGAT1	9913.ENSBTAP00000025329	9913.ENSBTAP00000037256	0	0	0	0	0	0	0.95	0.145	0.95
LOC618076	PPAP2C	9913.ENSBTAP00000026848	9913.ENSBTAP00000000955	0	0	0	0	0	0	0.94	0.083	0.945
LOC618076	DGAT2	9913.ENSBTAP00000026848	9913.ENSBTAP00000001536	0	0	0	1	0	0	0.9	0.531	0.902
LOC618076	PPAP2B	9913.ENSBTAP00000026848	9913.ENSBTAP00000015460	0	0	0	0	0	0	0.94	0.083	0.945
LOC618076	PPAP2A	9913.ENSBTAP00000026848	9913.ENSBTAP00000044571	0	0	0	0	0	0	0.94	0.083	0.945
LOC618076	DGAT1	9913.ENSBTAP00000026848	9913.ENSBTAP00000037256	0	0	0	0	0.1	0	0.9	0.458	0.954
LOC618076	PPAPDC1B	9913.ENSBTAP00000026848	9913.ENSBTAP00000039585	0	0	0	0	0	0	0.97	0.083	0.968
LOC618076	PPAPDC1A	9913.ENSBTAP00000026848	9913.ENSBTAP00000042923	0	0	0	0	0	0	0.97	0.083	0.968
LOC785379	DGAT2	9913.ENSBTAP00000056386	9913.ENSBTAP00000001536	0	0	0	1	0	0	0.9	0.328	0.91
LOC785379	DGAT1	9913.ENSBTAP00000056386	9913.ENSBTAP00000037256	0	0	0	0	0.1	0	0.9	0.345	0.952
LOC785379	PPAPDC1B	9913.ENSBTAP00000056386	9913.ENSBTAP00000039585	0	0	0	0	0.1	0	0.94	0.199	0.949
LOC785379	PPAPDC1A	9913.ENSBTAP00000056386	9913.ENSBTAP00000042923	0	0	0	0	0.1	0	0.94	0.199	0.949
LPIN1	PPAP2C	9913.ENSBTAP00000040997	9913.ENSBTAP00000000955	0	0	0	0	0	0	0.8	0.582	0.916
LPIN1	DGAT2	9913.ENSBTAP00000040997	9913.ENSBTAP00000001536	0	0	0	0	0.1	0	0.73	0.78	0.941
LPIN1	DGAT1	9913.ENSBTAP00000040997	9913.ENSBTAP00000037256	0	0	0	0	0.1	0	0.75	0.841	0.96
LPIN2	DGAT2	9913.ENSBTAP00000015997	9913.ENSBTAP00000001536	0	0	0	0	0.1	0	0.73	0.721	0.925
LPIN2	DGAT1	9913.ENSBTAP00000015997	9913.ENSBTAP00000037256	0	0	0	0	0.1	0	0.75	0.664	0.916
LPIN3	DGAT1	9913.ENSBTAP00000012081	9913.ENSBTAP00000037256	0	0	0	0	0.1	0	0.75	0.599	0.904
LPPR2	DGAT2	9913.ENSBTAP00000043142	9913.ENSBTAP00000001536	0	0	0	0	0	0	0.95	0.083	0.947
LPPR2	MOGAT1	9913.ENSBTAP00000043142	9913.ENSBTAP00000011091	0	0	0	0	0	0	0.9	0.083	0.908
LPPR2	DGAT1	9913.ENSBTAP00000043142	9913.ENSBTAP00000037256	0	0	0	0	0	0	0.95	0.145	0.95
MOGAT1	PPAP2C	9913.ENSBTAP00000011091	9913.ENSBTAP00000000955	0	0	0	0	0.1	0	0.96	0.702	0.989
MOGAT1	LOC513388	9913.ENSBTAP00000011091	9913.ENSBTAP00000025329	0	0	0	0	0	0	0.9	0.083	0.908

MOGAT1	LPPR2	9913.ENSBTAP00000011091	9913.ENSBTAP00000043142	0	0	0	0	0	0	0.9	0.083	0.908
MOGAT1	DGAT1	9913.ENSBTAP00000011091	9913.ENSBTAP00000037256	0	0	0	0	0.1	0	0.65	0.83	0.956
MOGAT1	PPAP2B	9913.ENSBTAP00000011091	9913.ENSBTAP00000015460	0	0	0	0	0	0	0.96	0.148	0.968
MOGAT1	PPAP2A	9913.ENSBTAP00000011091	9913.ENSBTAP00000044571	0	0	0	0	0	0	0.96	0.512	0.982
MOGAT1	PPAPDC1A	9913.ENSBTAP00000011091	9913.ENSBTAP00000042923	0	0	0	0	0.1	0	0.98	0.199	0.986
MOGAT1	PPAPDC1B	9913.ENSBTAP00000011091	9913.ENSBTAP00000039585	0	0	0	0	0.1	0	0.98	0.199	0.986
PNPLA2	DGAT2	9913.ENSBTAP00000029866	9913.ENSBTAP00000001536	0	0	0	0	0.1	0	0.8	0.826	0.965
PNPLA2	DGAT1	9913.ENSBTAP00000029866	9913.ENSBTAP00000037256	0	0	0	0	0.1	0	0.8	0.813	0.963
PPAP2A	DGAT2	9913.ENSBTAP00000044571	9913.ENSBTAP00000001536	0	0	0	0	0	0	0.99	0.383	0.996
PPAP2A	MOGAT1	9913.ENSBTAP00000044571	9913.ENSBTAP00000011091	0	0	0	0	0	0	0.96	0.512	0.982
PPAP2A	LOC618076	9913.ENSBTAP00000044571	9913.ENSBTAP00000026848	0	0	0	0	0	0	0.94	0.083	0.945
PPAP2A	DGAT1	9913.ENSBTAP00000044571	9913.ENSBTAP00000037256	0	0	0	0	0	0	0.99	0.349	0.996
PPAP2B	DGAT2	9913.ENSBTAP00000015460	9913.ENSBTAP00000001536	0	0	0	0	0	0	0.99	0.397	0.996
PPAP2B	MOGAT1	9913.ENSBTAP00000015460	9913.ENSBTAP00000011091	0	0	0	0	0	0	0.96	0.148	0.968
PPAP2B	LOC618076	9913.ENSBTAP00000015460	9913.ENSBTAP00000026848	0	0	0	0	0	0	0.94	0.083	0.945
PPAP2B	DGAT1	9913.ENSBTAP00000015460	9913.ENSBTAP00000037256	0	0	0	0	0	0	0.99	0.145	0.994
PPAP2C	ENSBTAG00000037483	9913.ENSBTAP00000000955	9913.ENSBTAP00000049702	0	0	0	0	0.1	0	0.86	0.396	0.915
PPAP2C	LPIN1	9913.ENSBTAP00000000955	9913.ENSBTAP00000040997	0	0	0	0	0	0	0.8	0.582	0.916
PPAP2C	LOC618076	9913.ENSBTAP00000000955	9913.ENSBTAP00000026848	0	0	0	0	0	0	0.94	0.083	0.945
PPAP2C	MOGAT1	9913.ENSBTAP00000000955	9913.ENSBTAP00000011091	0	0	0	0	0.1	0	0.96	0.702	0.989
PPAP2C	DGAT1	9913.ENSBTAP00000000955	9913.ENSBTAP00000037256	0	0	0	0	0	0	0.99	0.177	0.995
PPAP2C	DGAT2	9913.ENSBTAP00000000955	9913.ENSBTAP00000001536	0	0	0	0	0.1	0	0.99	0.56	0.997
PPAPDC1A	DGAT2	9913.ENSBTAP00000042923	9913.ENSBTAP00000001536	0	0	0	0	0.1	0	0.98	0.199	0.986
PPAPDC1A	MOGAT1	9913.ENSBTAP00000042923	9913.ENSBTAP00000011091	0	0	0	0	0.1	0	0.98	0.199	0.986
PPAPDC1A	LOC618076	9913.ENSBTAP00000042923	9913.ENSBTAP00000026848	0	0	0	0	0	0	0.97	0.083	0.968
PPAPDC1A	DGAT1	9913.ENSBTAP00000042923	9913.ENSBTAP00000037256	0	0	0	0	0	0	0.98	0.328	0.988
PPAPDC1A	LOC785379	9913.ENSBTAP00000042923	9913.ENSBTAP00000056386	0	0	0	0	0.1	0	0.94	0.199	0.949
PPAPDC1A	ENSBTAG00000037483	9913.ENSBTAP00000042923	9913.ENSBTAP00000049702	0	0	0	0	0.1	0	0.94	0.199	0.949
PPAPDC1B	DGAT2	9913.ENSBTAP00000039585	9913.ENSBTAP00000001536	0	0	0	0	0.1	0	0.98	0.306	0.988

PPAPDC1B	MOGAT1	9913.ENSBTAP00000039585	9913.ENSBTAP00000011091	0	0	0	0	0.1	0	0.98	0.199	0.986
PPAPDC1B	LOC618076	9913.ENSBTAP00000039585	9913.ENSBTAP00000026848	0	0	0	0	0	0	0.97	0.083	0.968
PPAPDC1B	DGAT1	9913.ENSBTAP00000039585	9913.ENSBTAP00000037256	0	0	0	0	0.1	0	0.98	0.328	0.989
PPAPDC1B	LOC785379	9913.ENSBTAP00000039585	9913.ENSBTAP00000056386	0	0	0	0	0.1	0	0.94	0.199	0.949
PPAPDC1B	ENSBTAG00000037483	9913.ENSBTAP00000039585	9913.ENSBTAP00000049702	0	0	0	0	0.1	0	0.94	0.199	0.949
RDH10	AWAT2	9913.ENSBTAP00000026830	9913.ENSBTAP00000016283	0	0	0	0	0.1	0	0.9	0.074	0.904
RDH10	CYP27C1	9913.ENSBTAP00000026830	9913.ENSBTAP00000052365	0	0	0	0	0	0	0.91	0.043	0.908
RDH10	DGAT1	9913.ENSBTAP00000026830	9913.ENSBTAP00000037256	0	0	0	0	0	0	0.9	0.226	0.919
RDH10	RETSAT	9913.ENSBTAP00000026830	9913.ENSBTAP00000029241	0.1	0	0	0	0.1	0	0.9	0.188	0.919
RDH10	CYP26A1	9913.ENSBTAP00000026830	9913.ENSBTAP00000028140	0	0	0	0	0	0	0.91	0.765	0.978
RDH10	RPE65	9913.ENSBTAP00000026830	9913.ENSBTAP00000041254	0	0	0	0	0	0	0.99	0.702	0.996
RDH11	AWAT2	9913.ENSBTAP00000002535	9913.ENSBTAP00000016283	0	0	0	0	0.1	0	0.9	0.049	0.903
RDH11	DGAT1	9913.ENSBTAP00000002535	9913.ENSBTAP00000037256	0	0	0	0	0	0	0.9	0.101	0.906
RDH11	CYP27C1	9913.ENSBTAP00000002535	9913.ENSBTAP00000052365	0	0	0	0	0	0	0.91	0.068	0.91
RDH11	RETSAT	9913.ENSBTAP00000002535	9913.ENSBTAP00000029241	0.1	0	0	0	0.1	0	0.9	0.302	0.929
RDH11	CYP26A1	9913.ENSBTAP00000002535	9913.ENSBTAP00000028140	0	0	0	0	0	0	0.91	0.378	0.94
RDH11	RPE65	9913.ENSBTAP00000002535	9913.ENSBTAP00000041254	0	0	0	0	0	0	0.92	0.662	0.971
RDH5	AWAT2	9913.ENSBTAP00000056512	9913.ENSBTAP00000016283	0	0	0	0	0	0	0.9	0	0.9
RDH5	DGAT1	9913.ENSBTAP00000056512	9913.ENSBTAP00000037256	0	0	0	0	0.1	0	0.9	0.119	0.91
RDH5	RPE65	9913.ENSBTAP00000056512	9913.ENSBTAP00000041254	0	0	0	0	0.1	0	0.99	0.937	0.999
RETSAT	RDH11	9913.ENSBTAP00000029241	9913.ENSBTAP00000002535	0.1	0	0	0	0.1	0	0.9	0.302	0.929
RETSAT	ADH4	9913.ENSBTAP00000029241	9913.ENSBTAP00000003769	0	0	0	0	0.1	0	0.92	0.16	0.929
RETSAT	ADH6	9913.ENSBTAP00000029241	9913.ENSBTAP00000009191	0	0	0	0	0.1	0	0.92	0	0.919
RETSAT	HSD17B6	9913.ENSBTAP00000029241	9913.ENSBTAP00000015564	0	0	0	0	0.1	0	0.9	0.158	0.914
RETSAT	AWAT2	9913.ENSBTAP00000029241	9913.ENSBTAP00000016283	0	0	0	0	0.1	0	0.9	0.069	0.904
RETSAT	ADH7	9913.ENSBTAP00000029241	9913.ENSBTAP00000020879	0	0	0	0	0.1	0	0.92	0.104	0.924
RETSAT	ADH5	9913.ENSBTAP00000029241	9913.ENSBTAP00000021304	0	0	0	0	0.1	0	0.92	0.366	0.946
RETSAT	DHRS4	9913.ENSBTAP00000029241	9913.ENSBTAP00000023493	0.1	0	0	0	0.1	0	0.9	0.422	0.944
RETSAT	SDR16C5	9913.ENSBTAP00000029241	9913.ENSBTAP00000024714	0.1	0	0	0	0.1	0	0.9	0.278	0.927

RETSAT	RDH10	9913.ENSBTAP00000029241	9913.ENSBTAP00000026830	0.1	0	0	0	0.1	0	0.9	0.188	0.919
RETSAT	CYP26A1	9913.ENSBTAP00000029241	9913.ENSBTAP00000028140	0	0	0	0	0.1	0	0.9	0.248	0.923
RETSAT	DHRS9	9913.ENSBTAP00000029241	9913.ENSBTAP00000055524	0	0	0	0	0.1	0	0.9	0	0.901
RETSAT	DGAT1	9913.ENSBTAP00000029241	9913.ENSBTAP00000037256	0	0	0	0	0.1	0	0.9	0.146	0.912
RETSAT	CYP27C1	9913.ENSBTAP00000029241	9913.ENSBTAP00000052365	0	0	0	0	0.1	0	0.9	0.307	0.929
RETSAT	DHRS3	9913.ENSBTAP00000029241	9913.ENSBTAP00000033930	0.1	0	0	0	0.1	0	0.9	0.373	0.938
RPE65	RDH11	9913.ENSBTAP00000041254	9913.ENSBTAP00000002535	0	0	0	0	0	0	0.92	0.662	0.971
RPE65	AWAT2	9913.ENSBTAP00000041254	9913.ENSBTAP00000016283	0	0	0	0	0	0	0.9	0.051	0.901
RPE65	RDH10	9913.ENSBTAP00000041254	9913.ENSBTAP00000026830	0	0	0	0	0	0	0.99	0.702	0.996
RPE65	DGAT1	9913.ENSBTAP00000041254	9913.ENSBTAP00000037256	0	0	0	0	0.1	0	0.9	0.354	0.936
RPE65	RDH5	9913.ENSBTAP00000041254	9913.ENSBTAP00000056512	0	0	0	0	0.1	0	0.99	0.937	0.999
SDR16C5	AWAT2	9913.ENSBTAP00000024714	9913.ENSBTAP00000016283	0	0	0	0	0.1	0	0.9	0.074	0.904
SDR16C5	CYP27C1	9913.ENSBTAP00000024714	9913.ENSBTAP00000052365	0	0	0	0	0	0	0.91	0.043	0.908
SDR16C5	RETSAT	9913.ENSBTAP00000024714	9913.ENSBTAP00000029241	0.1	0	0	0	0.1	0	0.9	0.278	0.927
SDR16C5	DGAT1	9913.ENSBTAP00000024714	9913.ENSBTAP00000037256	0	0	0	0	0.1	0	0.9	0.388	0.937
SDR16C5	CYP26A1	9913.ENSBTAP00000024714	9913.ENSBTAP00000028140	0	0	0	0	0	0	0.91	0.366	0.941
